# Development and Efficacy of an Electronic, Culturally Adapted Lifestyle Counseling Tool for Improving Diabetes-Related Dietary Knowledge: Randomized Controlled Trial Among Ethnic Minority Adults With Type 2 Diabetes Mellitus

**DOI:** 10.2196/13674

**Published:** 2019-10-16

**Authors:** Kathleen Abu-Saad, Havi Murad, Rivka Barid, Liraz Olmer, Arnona Ziv, Nuha Younis-Zeidan, Vered Kaufman-Shriqui, Michal Gillon-Keren, Shmuel Rigler, Yakir Berchenko, Ofra Kalter-Leibovici

**Affiliations:** 1 Cardiovascular Epidemiology Unit Gertner Institute for Epidemiology and Health Policy Research Ramat Gan Israel; 2 Biostatistics and Biomathematics Unit Gertner Institute for Epidemiology and Health Policy Research Ramat Gan Israel; 3 Israel Central Bureau of Statistics Jerusalem Israel; 4 Information and Computerization Unit Gertner Institute for Epidemiology and Health Policy Research Ramat Gan Israel; 5 Diet and Nutrition Service Unit for the Arab population in Sharon-Shomron District Clalit Health Services Arara Israel; 6 Department of Nutritional Sciences Ariel University Ariel Israel; 7 Institute of Endocrinology and Diabetes Schneider Children’s Medical Center Petah Tikva Israel; 8 Sharon-Shomron District Clalit Health Services Hadera Israel; 9 Department of Industrial Engineering and Management Ben-Gurion University of the Negev Beer Sheva Israel; 10 Sackler Faculty of Medicine Tel-Aviv University Tel Aviv Israel

**Keywords:** diabetes mellitus, type 2, diabetes-related dietary knowledge, lifestyle, software, culturally congruent care, ethnic minorities

## Abstract

**Background:**

Ethnic minority populations exhibit disproportionately high rates of type 2 diabetes mellitus (T2DM). Electronic health tools have the potential to facilitate the cultural adaptation and tailoring of T2DM education to improve the knowledge and management of diabetes mellitus (DM).

**Objective:**

This study aimed (1) to develop an adaptable Interactive Lifestyle Assessment, Counseling, and Education (I-ACE) software to support dietitian-delivered lifestyle counseling among low-socioeconomic status (SES) ethnic minority patients with T2DM and (2) to evaluate its effect on DM-related dietary knowledge and management compared with standard lifestyle advice (SLA) in a randomized controlled trial (RCT).

**Methods:**

The I-ACE software, developed in consultation with clinical dieticians, incorporates evidence-based dietary and physical activity (PA) recommendations and educational materials. The features and behavioral change techniques include quantitative lifestyle (dietary intake and PA) assessment and simulation, individually tailored education and recommendations, motivational interviewing, and goal setting. For the unblinded pilot RCT, 50 overweight or obese Arab adults (aged 40-62 years) with poorly controlled T2DM were recruited from primary care clinics and randomly assigned to receive 4 in-person, dietician-delivered counseling sessions over 6 months using either (1) the I-ACE tool (experimental arm) or (2) the SLA methods (comparison arm). All outcome assessments were face-to-face. DM-related dietary knowledge (primary outcome) was measured at baseline, 3, 6, and 12 months. Lifestyle and other parameters were measured before, during, and after the intervention. Multiple linear regression and repeated measures linear mixed models were used to compare the changes in study outcomes and explore time trends in between-group and within-group changes.

**Results:**

A total of 25 participants were enrolled in each arm, of whom 24 and 21 completed the final assessment of the primary outcome in the I-ACE and SLA arms, respectively. DM-related lifestyle knowledge increased more rapidly in the I-ACE arm than in the SLA arm (*P* value for study arm×time interaction=.02). Within the I-ACE arm, the mean (SE) differences in added sugar and dietary fiber intakes from baseline to 12 months were −2.6% (SE 1.0%) of total energy (*P*=.03) and 2.7 (SE 0.0) g/1000 kcal (*P*=.003), respectively. The odds of engaging in any leisure PA at 12 months tended to be higher in the I-ACE arm versus SLA arm, but did not reach statistical significance (odds ratio 2.8; 95% CI 0.7-11.6; *P*=.16). Both arms exhibited significant reductions in HbA_1c_ (*P* value for change over time <.001).

**Conclusions:**

The use of the I-ACE software in a 6-month, 4-session dietician-delivered lifestyle counseling intervention improved the efficiency of lifestyle education, compared with SLA, among low-SES, ethnic minority patients with T2DM. This pilot trial provides justification for conducting a large-scale trial to evaluate its effectiveness and applicability in routine clinical care among ethnically diverse populations.

**Trial Registration:**

ClinicalTrials.gov NCT01858506; https://clinicaltrials.gov/ct2/show/NCT01858506.

## Introduction

### Background

Diabetes mellitus (DM) is a progressive chronic disease that can result in serious short- and long-term complications. Patient self-management education and support are fundamental to improving DM management [1-6], and guidelines recommend that every person with DM receive self-management education in a format appropriate for the patient’s specific cultural, socioeconomic, literacy, and numeracy characteristics [2,3]. A growing number of self-management education programs for patients with DM are incorporating information technologies (IT) to improve their effectiveness and reach [7-9].

Nutrition therapy is one of the most challenging components of self-management for many patients with type 2 DM (T2DM). It is therefore particularly important that patients receive dietary education and collaborate with providers to develop individualized eating plans that are both implementable and sustainable and incorporate their preferences and needs [3]. Despite its critical role, few studies have focused on the nutritional education and counseling component of these DM self-management interventions [4-6,9-12] or reported their effectiveness in improving DM-specific dietary knowledge [7,8].

Patient-centered DM dietary education is especially critical for ethnic/racial minority populations, who often bear a disproportionately high burden of T2DM [2,13,14]. The standard dietary education and advice provided by mainstream health care services may not adequately address the daily challenges faced by minority patients as their cultural, social, and dietary norms and socioeconomic realities differ from that of the majority population. IT tools can provide new opportunities to make DM-related dietary education and counseling more relevant and individually tailored for patients [7,8]; however, few existing examples/initiatives have included sizeable proportions of ethnic minority patients [7,15]. There is a need to expand the evidence base for new digital health technologies that can address these needs [16] among the highest-risk, most vulnerable patient populations.

### Objectives

In this paper, we report the development of a dietician-operated, culturally adaptable Interactive Lifestyle Assessment, Counseling, and Education (I-ACE) software. We further report the results of a pilot randomized controlled trial (RCT) evaluating its effect, compared with standard lifestyle advice (SLA), on improving DM-related dietary knowledge, lifestyle behaviors, and glycemic control in a sample of low-socioeconomic status (SES) adults with T2DM from the Arab minority in Israel.

## Methods

### Design

This was an open, parallel-group, pilot RCT randomized controlled pilot trial testing the effect of a 6-month, dietician-delivered, face-to-face diabetes lifestyle (diet and physical activity [PA]) counseling program using the I-ACE software compared with SLA. The I-ACE software was adapted to provide culturally congruent lifestyle counseling to Arab adults with T2DM.

### Ethical Considerations

Ethics approval was obtained from the Helsinki committees of Sheba Medical Center and Clalit Health Services, and all participants provided written informed consent before enrollment. The study was registered at ClinicalTrials.gov (NCT01858506). The CONSORT eHealth checklist is provided in Multimedia Appendix 1.

### Study Population and Participants

The Arab minority in Israel is an indigenous population that accounts for 20% of the total population. It differs in language, culture, and religion from the majority Jewish population and, for the most part, resides in residentially segregated and economically deprived communities [17]. It is characterized by a lower SES and higher rates of T2DM, poor glycemic control, and diabetic complications, than the majority population [17-20].

A total of 50 eligible participants were recruited from the clinics of Clalit Health Services in 2 Arab towns. Inclusion criteria were: (1) age between 40 and 64 years, (2) diagnosis of T2DM, (3) having T2DM for ≤10 years, (4) body mass index (BMI) of 27 to 43 kg/m^2^, and (5) hemoglobin A_1c_ (HbA_1c_) between 8.0% and 11.3%. Participants were not eligible if they (1) were receiving short-acting insulin treatment, (2) had inadequate control of comorbid conditions, or (3) had factors that would limit adherence to interventions (eg, any medical or physical condition that prohibited participation in PA or following standard diets recommended for people with diabetes, pregnancy, uncontrolled psychiatric condition, significant cognitive impairment, or blindness).

Recruitment was conducted at the local Clalit Health Service clinics, in collaboration with the physicians of the potential participants. The lists of potential participants meeting the inclusion criteria were generated from Clalit electronic medical records and reviewed by the patients’ physicians to identify eligible candidates. Patients with an HbA_1c_ result measured more than 3 months before eligibility screening were requested to get the test repeated. Baseline evaluation (including initial lifestyle knowledge, dietary and PA assessments, and anthropometric measurements) were completed in the clinic before randomization.

Eligible participants were randomly assigned in 1:1 ratio either to the I-ACE or SLA study arm using a permuted block design, central computer–generated randomization process, with even-numbered blocks of 2 to 6 participants. The randomization was performed at the Gertner Institute. Allocation concealment was maintained until after the provision of informed consent and randomization. Although the intervention type was known to the participants, dieticians, and study coordinator, the study statisticians were blinded to group allocation until the primary study outcomes analyses were completed. Group assignment was masked from all health care service providers other than the dietician.

The recruitment ran from August 2014 to January 2015. The participant follow-up and data collection from the electronic medical records was completed in March 2016. The pilot trial ended, according to the protocol, after all the participants had been followed up for 1 year.

### Sample Size Considerations

The sample size calculation was based upon the reported differences in the nutritional knowledge change (percentage of correct responses) between the intervention and comparison groups in 2 nutritional educational interventions, which ranged from 8.9% to 11.5% [21,22]. A sample of 50 participants was needed to provide 90% power to detect a statistically significant difference of this magnitude at the 5% level using a 2-sided test between the experimental and comparison groups, allowing for a dropout rate of 10%.

### Interventions

The study included 2 active intervention arms: (1) the I-ACE experimental arm and (2) the SLA comparison arm. The experimental arm of the intervention used the I-ACE software.

#### Information Technology Tool Description

The I-ACE software was designed for use by dieticians to support and enrich a patient-centered clinical lifestyle counseling process. It is a multifeatured tool that supports collecting data on habitual dietary and PA behaviors and using these data to calculate actionable, graphically displayed summary measures (eg, average daily or weekly food/nutrient intakes and PA). Additional I-ACE features support the dietician-patient team in building and tracking an individually tailored healthy lifestyle program.

I-ACE was designed with a Windows (Microsoft Corporation) platform. It has system tables that incorporate food and nutrient databases and evidence-based age-specific, sex-specific, or health status–specific goal packages [3,23-25]. It makes extensive use of embedded graphics, enables the uploading and modification of pictures/infographics/educational materials, and provides graphic presentation of the patient’s progress over time. Experienced clinical dieticians (NYZ, MG, and VKS) provided input and feedback on the software’s professional content and clinical use features during the development phase. The dieticians in this study, who were the primary users of I-ACE, were computer-literate and routinely used administrative computer programs in their clinical practice.

In addition to the tools for supporting the clinical counseling sessions, I-ACE has administrative-level tools, which (1) can adapt the counseling support tools for use among patients/clients of different (and multiple) ethnic, age, life stage, and/or health status groups, (2) can document all phases of the consultation process for quality control and effectiveness assessment, and (3) has data importing, exporting, and reporting features to support institutional oversight, evaluation, and research activities.

I-ACE enriches the standard approach to dietary counseling in several ways. It uses a food frequency questionnaire (FFQ) and PA questionnaire to systematically document and quantify habitual lifestyle behaviors (for further details on the questionnaires, see the Cultural Adaptation of the Information Technology Tool section) [26,27]. These patient-reported data are summarized and compared with evidence-based food and nutrient intake goals, modeled by the consultant study dieticians on a Mediterranean diet [24,25] and adapted for people with diabetes. These tools are used to individually tailor, focus, and prioritize the educational and counseling processes through identifying lifestyle behaviors that need improvement as targets for education and behavioral change. The counseling component uses Pareto [28] and motivational interviewing [29] principles to set personalized goals, identify the minimal amount of change needed to achieve the maximal impact, simulate changes, and document the patients’ willingness to change. Agreed-upon changes are summarized in a take-home report for the patient and followed up in subsequent counseling sessions. These features are further described along with sample screenshots in Multimedia Appendix 2.

The software includes embedded, modifiable lifestyle educational content based on the published standards of care for medical nutrition therapy for patients with T2DM [3,23] in an easy-to-understand format [30]. It conveys a general and applied understanding of the difference between complex and simple carbohydrates, carbohydrate exchange portions, the glycemic properties of foods, different types of fats (saturated, unsaturated, and trans), optimal sources of dietary fiber and protein, and nutrient-dense versus nutrient-poor foods. The PA content is based upon the World Health Organization’s recommendation of at least 150 min/week of moderate-intensity aerobic PA distributed over most days of the week [3,23].

In summary, the software augments and structures the standard approach to lifestyle counseling by providing systematic documentation and quantification of lifestyle data and a graphic interface for education, goal setting, problem solving, and individual tailoring, many aspects of which are modifiable. These features provide new tools to support and expand the active participation of the counselee in making a behavior change plan that suits his/her life.

#### Cultural Adaptation of the Information Technology Tool

I-ACE provides language congruence through its multilanguage capacity (stage 1: English, Arabic, and Hebrew). In addition, multiple components of the software were culturally adapted for this study. The food database, FFQ, and intervention approach were based on prior epidemiological and interventional research. The I-ACE FFQ included approximately 90 food items that accounted for over 80% of the total energy intake of Arab participants in an earlier, population-based dietary assessment we conducted [26]. In addition, it allowed for other food items to be added from the embedded food database, with 500+ items, developed for the Jewish and Arab populations in Israel [26]. The PA questionnaire was also previously used in our epidemiological research with the Arab population [26,27]. This study intervention was built upon the cultural adaptations that were made for our prior interventional study among Arab women [27]. Those adaptations were directed by the study’s Arab coinvestigators and its dieticians, all of whom were from the local Arab community. In addition, focus groups were conducted with local Arab women to obtain their input on cultural and practical aspects of the intervention [27]. For this study, the Arab study investigator and consultant dietician (NYZ) provided input on the adaptation of the software’s embedded food photos/graphics and educational materials to reflect the local food customs and cultural norms (see Multimedia Appendix 2 for screen shots of culturally adapted educational materials). The software and cultural content underwent further iterative modifications based on the feedback from the Arab study dieticians. Translation of the infographics and software screens into Arabic was done by a professional translator from the local Arab community and reviewed by the Arab study investigator and dieticians.

#### Standard Lifestyle Counseling

The comparison SLA arm of the intervention received standard lifestyle counseling as provided by Clalit Health Service dieticians, using existing tools (which did not support quantified dietary assessment) and standard educational materials in Arabic.

#### Intervention Protocol

Both study arms received 4 individual dietary counseling sessions in the local clinic (in the first, second, third, and sixth months after randomization) and 1 group T2DM self-management session led by a nurse. The first and final dietary counseling sessions (both of which included full dietary assessments) were each approximately 30 to 45 min long for both arms. The median length of the second and third sessions was 17 min each (interquartile range [IQR] 11-26) in the experimental arm, whereas the follow-up visits in the SLA arm were each allotted 15 min, in keeping with the current practice in standard care. Both study arms received very active outreach to encourage adherence to the study protocol and assessments. This differs from standard care, in which intensive, active outreach is not the norm.

We used the same dieticians to provide counseling to both study arms to exclude the possibility of the differences between the groups occurring because of the differences between the dieticians, rather than the intervention type. Most of the intervention was delivered by a single dietician. The dieticians received 2 sessions of 6-hour training for using the I-ACE software before the RCT commenced and had ongoing oversight/support from the principal investigator (KA) and main consultant clinical dietician and coinvestigator (NYZ).

Multimedia Appendix 3 provides a summary of the intervention delivery by study arm.

Intervention adherence in both arms was measured by the attendance of the counseling sessions. The adherence to the use of I-ACE in the dietary counseling sessions in the experimental study arm was measured by checking for the existence of a visit record, including the documented use of the assessment and simulation features.

### Outcomes

#### Primary Outcome

The primary outcome was the diabetes-related dietary knowledge, measured at baseline, 3, 6, and 12 months.

#### Secondary Outcomes

Secondary outcomes included the (1) dietary intake, measured at baseline and 12 months for all participants (and additionally at 2, 3, and 6 months for participants in the I-ACE arm), (2) leisure PA (LPA), measured at baseline and 12 months, (3) anthropometric measurements (weight and waist circumference [WC]), measured at baseline, 2, 3, 6, and 12 months, and (4) HbA_1c_, measured before baseline and at 3, 6, and 12 months.

### Measures

#### Primary Outcome (Diabetes Mellitus–Related Dietary Knowledge)

We were not able to find any questionnaires in the literature solely dedicated to DM-related dietary knowledge; however, we did find several general DM knowledge questionnaires that had dietary questions [31-33]. We identified 9 questions from these DM knowledge questionnaires and adapted them to the dietary context of the target population (see examples of the adaptation in Multimedia Appendix 4). We added 2 questions probing the exchange portion sizes and/or the limitations on the intake of certain foods for people with diabetes. Diabetes-related dietary knowledge was assessed as the percentage of correct answers to these questions. An English translation of the DM-related lifestyle knowledge questionnaire is presented in Multimedia Appendix 5. The 2 LPA knowledge questions were taken from existing questionnaires, without need for adaptation [31,34]. The questionnaire was administered at baseline, after the counseling sessions at 3 and 6 months, and at 12 months.

#### Secondary Outcomes

#### Dietary Intake

Habitual dietary intake was measured using the computerized I-ACE FFQ, which was based upon FFQs developed by our research group for use among the Arab population in Israel [26,27]. The FFQ was administered to all participants at baseline and 12 months. As the FFQ was used in the experimental arm as a part of the I-ACE counseling approach to track dietary change at each session, the experimental arm also had dietary intake data at 2, 3, and 6 months.

#### Leisure Physical Activity

Habitual LPA (including the type of activity, frequency, and duration) data were collected using a questionnaire previously used in our research among this population [26,27]. The PA questionnaire was administered at baseline and 12 months.

#### Anthropometric Measurements

At baseline, weight and height (without shoes, in lightweight clothing) were measured with clinic scales and stadiometers and WC was measured at the midspace between the lowest costal margin and the iliac crest with ergonomic circumference measuring tapes (Seca Medical Measurement Systems and Scales). Weight and WC measurements were repeated at each study visit (2, 3, 6, and 12 months). Weight and height were used to calculate BMI.

#### Hemoglobin A1c

HbA_1c_ test results were extracted from the electronic medical record for all potential participants before the recruitment to determine eligibility. During the study, the participants were requested to do HbA_1c_ tests every 3 months and all HbA_1c_ test results during the 12-month study period were extracted for the final analysis.

#### Additional Covariates

Demographic (eg, age, marital status, and education) and health status data were collected at baseline. General DM knowledge was evaluated via the Spoken Knowledge in Low Literacy in Diabetes scale (SKILLD) [34], which was administered at baseline and at 12 months. Information on the pharmacological diabetes management regimen was collected from the electronic medical records at baseline and at 12 months.

#### Participant/User Satisfaction

We developed a set of questions to elicit the participants’ perspectives on the utility of the counseling (in terms of improving their understanding and adherence), satisfaction with the counseling, and for those in the I-ACE arm, satisfaction with the software. We also obtained feedback from the study dietician at the end of the intervention on her experience using the software.

All measures were collected by the study coordinator (face-to-face or by phone), aside from the FFQ, which was administered (face-to-face) by the study dietician.

### Analytical Methods

For the primary outcome of DM-related dietary knowledge, the mean (SD) values were reported. A linear mixed regression model for repeated measures was used to evaluate the change in dietary knowledge over time, with an interaction term to determine whether the study groups differed from each other over time. There were missing data in this outcome at the different evaluation periods (34% [17/50] at 3 months, 48% [24/50] at 6 months, and 10% [5/50] at 12 months); however, as there were no missing observations at baseline, we compared the mean baseline DM-related dietary knowledge scores in participants with observed values and those with missing values at each evaluation period. Similar averages imply noninformative missingness (ie, missing at random), supporting the appropriateness of using a linear mixed model for repeated measures with maximum likelihood for this analysis.

For continuous secondary outcomes (eg, food and nutrient intakes, BMI, WC, and HbA_1c_), mean (SD) values were reported. Multiple linear regression models were used to test for the differences between the study groups at 12 months for changes in lifestyle behaviors. Linear mixed regression models for repeated measures were used to evaluate within-person change over time in dietary intakes in the I-ACE arm as repeated measures from each counseling session were available. WC and BMI, which had repeated measurements in both study arms, were evaluated with linear mixed regression models for repeated measures, with interaction terms for study group by time. For the repeated outcome measure HbA_1c_, we applied a linear mixed model on the log scale (owing to its non-normal distribution), including a random intercept and potential fixed effects (gender, study arm, age, and baseline DM drug therapy), using the *nlme* package in R. We considered several functions for modeling the effect of time at knots around 6 months as HbA_1c_ was not measured exactly at 6 months, including (1) 2 intervals with the cut-point of 6 months, (2) 2 intervals with the cut-point of 8 months, (3) natural cubic splines with 1 knot, and (4) natural cubic splines with 2 knots. We compared the mean square error of the models resulting from the different functions using cross-validation. The predicted values for a typical subject from each study arm were presented in a graph for each time. The expected difference in HbA_1c_ from baseline to 6 months and 12 months and their 95% CIs were calculated from the chosen model.

For binary secondary outcomes (eg, any LPA and recommended LPA level), raw count (number, %) was reported. Multiple logistic regression models were used to test for the differences between the study groups in these outcomes at 12 months.

Sex was forced into all multivariable models. Other key variables (age, study town, DM therapy, SKILLD score, and education) were entered into the models and only those with *P*<.10 were retained. Given the small sample size, it was important to keep the models as parsimonious as possible.

All analyses were performed using SAS version 9.4 SAS Institute, except for the HbA_1c_ repeated measures outcome, which was performed with the open-source statistical software platform R [35]. Simple tabulation and narrative description were used to report the participant and dietician feedbacks on the utility and satisfaction with the counseling and the I-ACE software.

## Results

### Participant Flow

**Figure 1:**
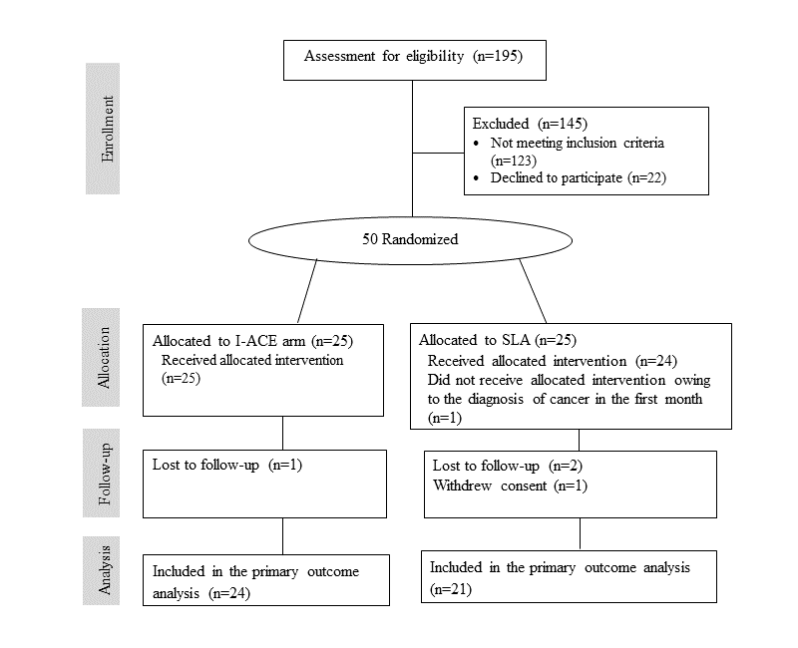
Screening, randomization, and completion of follow-up flow chart for the pilot trial of a culturally-adapted lifestyle counseling software among Arab adults with T2DM. T2DM: Type 2 diabetes mellitus; I-ACE: Interactive lifestyle Assessment, Counseling and Education; SLA: Standard Lifestyle Advice.

The participant flow chart is presented in Figure 1. A total of 195 potential participants were identified from Clalit electronic medical records, of whom 123 did not meet the inclusion criteria, 22 refused to participate, and 50 were randomized (25 to the I-ACE arm and 25 to the SLA arm). All those randomized to the I-ACE arm received the allocated intervention (n=25), and all but 1 participant (who was diagnosed with cancer in the first month after randomization) assigned to the SLA arm received the allocated intervention (n=24). One patient with confirmed diabetes and baseline HbA_1c_ of 6.1% was included in the study by mistake and randomized to the I-ACE arm. This patient was included in the data analysis. Furthermore, 3 participants, 1 in the I-ACE arm and 2 in the SLA arm, were lost to follow-up and 1 participant in the SLA arm withdrew consent.

### Missing Data

Data on the primary outcome (DM-related dietary knowledge) were collected from all 50 participants at baseline (25 in the I-ACE arm and 25 in the SLA arm), but was missing for 17 participants (34%) at the 3-month evaluation (7 in the I-ACE arm and 10 in the SLA arm), 24 participants (48%) at the 6-month evaluation (13 in the I-ACE arm and 11 in the SLA arm), and 5 participants (10%) at the 12-month evaluation (1 from the I-ACE arm and 4 from the SLA arm). To check whether the missing data pattern was informative, we compared the average baseline DM-related dietary knowledge score of participants with observed values to those with missing values at each evaluation period. We did not find significant differences at any of the periods, implying noninformative missingness (see Multimedia Appendix 6).

Information on between-group change in dietary behaviors from baseline to 12 months was missing for 9 participants (4 from the I-ACE arm and 5 from the SLA arm). The analysis of within-group change in dietary behaviors across the intervention counseling visits included all I-ACE participants with more than 1 counseling visit (n=25). The LPA outcomes were missing for 5 participants (1 from the I-ACE arm and 4 from the SLA arm). All 50 participants (25 in each arm) were included in the WC analysis and HbA_1c_ analyses. Counseling satisfaction and utility questionnaires were missing for 5 participants (2 from the I-ACE arm and 3 from the SLA arm). All analyses were conducted according to the originally assigned study groups.

### Baseline Data

Table 1 presents the baseline characteristics of the participants, none of which differed significantly by study arm. The average age of the participants at baseline was 53 years, and over 60% were treated with basal insulin, with or without additional oral hypoglycemic therapy. The mean baseline HbA_1c_ was above 9.0% in both study arms.

Table 2 presents the baseline diabetes knowledge and lifestyle behavior data. Participants did not differ on the overall general diabetes knowledge score as measured by the SKILLD scale, which was below 50 on a scale of 100. There were also no significant differences in baseline lifestyle (diet and PA) knowledge or behavior variables by study arm. Participants had suboptimal intakes of dietary fiber, vegetables, and whole grains and very low participation in LPA.

To construct the DM-related lifestyle knowledge score, we excluded the knowledge questions for which ≥85% of participants gave correct answers at baseline, to focus the score on items in need of and amenable to modification by the intervention. The items included in the score are noted in Table 2.

**Table 1:** Baseline characteristics of 50 Arab participants with type 2 diabetes mellitus in the pilot trial of a culturally adapted lifestyle counseling information technology tool by study group.

Participant characteristics		Total (N=50)	Study arm		*P* value
			I-ACE^a^ (n=25)	SLA^b^ (n=25)	
Sex (female), n (%)		29 (58)	17 (68)	12 (48)	.15
Age (years), mean (SD)		53.0 (7.6)	52.8 (7.9)	53.2 (7.4)	.87
Married, n (%)		43 (86)	21 (84)	22 (88)	.68
Education (years), mean (SD)		10.8 (3.7)	10.6 (4.2)	11.0 (3.2)	.71
Employed, n (%)		19 (38)	9 (36)	10 (40)	.77
**Study town, n (%)**					.57
	1	26 (48)	12 (48)	14 (56)	—^c^
	2	24 (52)	13 (52)	11 (44)	—
Dyslipidemia^d^, n (%)		41 (82)	21 (84)	20 (80)	.71
Hypertension^d^, n (%)		22 (44)	10 (40)	12 (48)	.57
Cardiovascular disease^d^, n (%)		11 (22)	4 (16)	7 (28)	.31
Number of chronic conditions^d^, mean (SD)		3.8 (1.5)	3.9 (1.6)	3.7 (1.5)	.64
Physical disability, n (%)		12 (24)	6 (24)	6 (24)	>.99
Age at DM^e^ diagnosis (years), mean (SD)		43.8 (7.7)	43.2 (7.6)	44.5 (7.8)	.56
DM duration (years), mean (SD)		9.2 (5.4)	9.6 (5.8)	8.7 (5.0)	.55
**DM therapy, n (%)**					.88
	Diet	1 (2)	0 (0)	1 (4)	—
	OHT^f^	18 (36)	10 (40)	8 (32)	—
	Basal insulin	2 (4)	1 (4)	1 (4)	—
	Basal insulin+OHT	29 (58)	14 (56)	15 (60)	—
Hemoglobin A_1c_ at baseline (%), mean (SD)		9.2 (1.1)	9.1 (1.3)	9.3 (1.0)	.57
Body mass index (kg/m^2^), mean (SD)		33.0 (4.1)	33.9 (4.3)	32.1 (3.7)	.17
Waist circumference (cm), mean (SD)		108.2 (9.8)	108.4 (9.9)	108.1 (9.9)	.90
Know last hemoglobin A_1c_ test result, n (%)		39 (78)	21 (84)	18 (72)	.31
**Self-blood glucose monitoring frequency, n (%)**					.57
	Daily	13 (26)	8 (32)	5 (20)	—
	Several times a week	12 (24)	6 (24)	6 (24)	—
	At least once a month but less than weekly	12 (24)	4 (16)	8 (32)	—
	Rarely/never	12 (24)	6 (24)	6 (24)	—

^a^I-ACE: Interactive Lifestyle Assessment, Counseling, and Education.

^b^SLA: standard lifestyle advice.

^c^Not applicable.

^d^On the basis of self-reported physician diagnosis or medical therapy.

^e^DM: diabetes mellitus.

^f^OHT: oral hypoglycemic therapy.

**Table 2:** Baseline levels of correct diabetes mellitus–related knowledge and lifestyle behaviors among 50 Arab patients with type 2 diabetes mellitus in the Interactive Lifestyle Assessment, Counseling, and Education pilot trial by study group.

Diabetes-related knowledge (% correct) and lifestyle behaviors			Total (N=50)	Study arm		*P* value
				I-ACE^a^ (N=25)	SLA^b^ (N=25)	
**SKILLD^c^ general DM^d^ knowledge**						
	Signs of high blood sugar, n (%)		14 (28)	9 (36)	5 (20)	.21
	Signs of low blood sugar, n (%)		10 (20)	3 (12)	7 (28)	.16
	What to do if blood sugar level is too low, n (%)		4 (8)	0 (0)	4 (16)	.04
	Frequency of self-foot check, n (%)		21 (42)	10 (40)	11 (44)	.78
	Rationale for self-foot check, n (%)		29 (58)	14 (56)	15 (60)	.78
	Frequency and rationale for having eyes checked, n (%)		33 (66)	19 (76)	14 (56)	.14
	Normal fasting blood sugar level, n (%)		39 (78)	19 (76)	20 (80)	.73
	Normal hemoglobin A_1c_ level, n (%)		30 (60)	16 (64)	14 (56)	.56
	Frequency and length of LPA^e^ per week, n (%)		18 (36)	10 (40)	8 (32)	.56
	Long-term complications of uncontrolled DM, n (%)		44 (88)	22 (88)	22 (88)	>.99
	SKILLD score, mean (SD)		48.4 (20.6)	48.8 (19.4)	48.0 (22.2)	.89
**DM-related dietary knowledge**						
	**Know limitations^f^ on the consumption of:**					
		Honey^g^, n (%)	6 (12)	4 (16)	2 (8)	.38
		Dates^g^, n (%)	35 (70)	20 (80)	15 (60)	.12
		Yogurt/buttermilk^g^, n (%)	27 (54)	13 (52)	14 (56)	.78
		Cola (regular and nondiet)^g^, n (%)	1 (2)	0 (0)	1 (4)	.31
		Vegetable salad, n (%)	46 (92)	24 (96)	22 (88)	.30
		Rice, n (%)	49 (98)	25 (100)	24 (96)	.31
		Pita/bread, n (%)	45 (90)	23 (92)	22 (88)	.64
		Cookies (nondiet)^g^, n (%)	15 (30)	5 (20)	10 (40)	.12
		Grapes^g^, n (%)	31 (62)	18 (72)	13 (52)	.15
		Fruit juice^g^, n (%)	3 (6)	0 (0)	3 (12)	.07
		Special (sugar-free) food products for diabetics, n (%)	43 (86)	23 (92)	20 (80)	.22
	Identify food highest in carbohydrates^g^, n (%)		39 (78)	18 (72)	21 (84)	.31
	Identify healthy fat source^g^, n (%)		24 (48)	11 (44)	13 (52)	.57
	**Identify standard exchange portion for, n (%)**					
		Pita^g^	4 (8)	2 (8)	2 (8)	>.99
		Rice^g^	7 (14)	3 (12)	4 (16)	.68
		Apple^g^	38 (76)	20 (80)	18 (72)	.51
		Yogurt/buttermilk^g^	39 (78)	21 (84)	18 (72)	.31
		Dried dates^g^	16 (32)	8 (32)	8 (32)	>.99
	Identify food that raises blood sugar the fastest^g^, n (%)		31 (62)	17 (68)	14 (56)	.38
	Identify food that raises blood sugar most slowly^g^, n (%)		20 (40)	11 (44)	9 (36)	.56
	Identify best food/drink to treat hypoglycemia^g^, n (%)		29 (58)	13 (52)	16 (64)	.39
	Know the effect of physical activity on blood sugar, n (%)		49 (98)	25 (100)	24 (96)	.31
	Special (sugar-free) food products not essential to glycemic control^g^, n (%)		30 (60)	15 (60)	15 (60)	>.99
	Limiting salt intake reduces blood pressure, n (%)		45 (90)	21 (84)	24 (96)	.16
	Reducing SFA intake reduces cardiovascular disease risk, n (%)		47 (94)	24 (96)	23 (92)	.55
	All questions (% correct answers), mean (SD)		56.7 (9.0)	57.5 (10.2)	55.9 (7.6)	.51
	DM-lifestyle knowledge score (% correct answers), mean (SD)		44.4 (10.9)	45.3 (12.1)	43.5 (9.6)	.58
**Dietary behaviors, mean (SD)**						
	Added sugar (% of total energy)		5.4 (0.1)	5.5 (0.1)	5.4 (0.1)	.99
	Dietary fiber (g/1000 kcal)		9.6 (2.5)	9.7 (2.6)	9.5 (2.4)	.89
	Fruit (portions/day)		3.0 (1.7)	3.0 (1.7)	2.9 (1.7)	.66
	Vegetables (portions/day)		3.8 (2.2)	3.4 (1.9)	4.2 (2.4)	.31
	Whole grains (portions/day)		2.2 (2.6)	2.2 (2.7)	2.2 (2.5)	.86
**LPA^f^ behaviors, n (%)**						
	Any LPA		9 (18)	5 (20)	4 (16)	>.99
	≥150 min LPA/week		4 (8)	2 (8)	2 (8)	>.99

^a^I-ACE: Interactive Lifestyle Assessment, Counseling, and Education.

^b^SLA: standard lifestyle advice.

^c^SKILLD: Spoken Knowledge in Low Literacy in Diabetes.

^d^DM: diabetes mellitus.

^e^LPA: leisure physical activity.

^f^From the following categories: completely forbidden, only to be consumed to treat a hypoglycemic episode, can be consumed in limited amount, and can be consumed without limitation.

^g^Items included in the diabetes mellitus–related dietary knowledge score.

### Study Process

The mean (SD) number of counseling sessions attended (out of a maximum of 4) did not differ between the I-ACE and SLA arms (3.44 [SD 0.96] vs 2.92 [SD 1.22] respectively; *P*=.10) Over two-thirds (68%) of the participants in the I-ACE arm attended all 4 counseling sessions and another 16% attended 3 sessions, representing a high adherence of 84%. In the SLA arm, 48% and 16% of the participants attended 4 and 3 counseling sessions, respectively, totaling 64% with high adherence. Session attendance adherence tended to be higher among women than men, but this varied by study arm. Similar proportions of men and women attended at least 3 sessions in the I-ACE arm (88% vs 82%, respectively; *P*=.46); whereas the proportion tended to be lower for men than women in the SLA arm (54% vs 75%, respectively; *P*=.03; see Multimedia Appendix 7 for the number of visits and qualitative information about adherence barriers). The digital visit records saved in the I-ACE software for the experimental arm indicated the intervention adherence (ie, the use of the software for assessment and counseling) in 100% of first visits and 85% of follow-up visits.

### Outcomes

**Figure 2:**
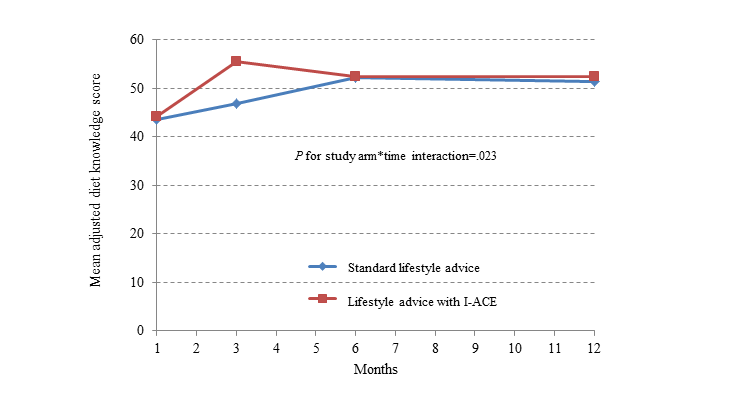
Change in DM-related lifestyle knowledge score during intervention (up to 6 months) and follow-up (up to 12 months) among 50 Arab patients with type 2 diabetes mellitus in the I-ACE pilot trial by study arm. Results of a linear mixed regression model for repeated measures with a time*study arm interaction, controlling for sex, educational level, and number of study dietary counseling visits. DM: diabetes mellitus; I-ACE: Interactive lifestyle Assessment.

The primary study outcome, change in DM-related lifestyle knowledge from baseline, differed between the study arms over time (Figure 2; *P* value for study arm×time interaction=.02). Multimedia Appendix 8 shows the results of the linear mixed model for repeated measures testing the within-group and between-group differences in the knowledge score from baseline to 3, 6, and 12 months. In the I-ACE arm, the mean knowledge score was significantly higher at 3 months than the baseline score and a significant difference was maintained through 12 months. In the SLA arm, the accrual of knowledge occurred more slowly and did not differ significantly from baseline until 6 months. The difference in the slope of the knowledge scores between the study arms was significant at 3 months (higher for I-ACE, *P*=.03). From the sixth month onward, both groups had approximately equal levels of DM-related lifestyle knowledge, which remained significantly higher at 12 months than their mean baseline scores. The increase in knowledge was significantly greater for women than for men (mean [SE] difference=5.14 [SE 2.28]; *P*=.03), whereas, the median (IQR) educational level was lower for women than for men (9 [IQR 8-12] years vs 12 [IQR 10-15] years, respectively; *P*=.049).

Multimedia Appendix 9 presents the change in lifestyle behaviors from baseline to 12 months. With regard to the dietary behaviors recommended for diabetes management (eg, adequate consumption of nonstarchy vegetables, whole grains, and dietary fiber and limited consumption of fruit and added sugar), the I-ACE arm exhibited a significant reduction in added sugar intake and a significant increase in dietary fiber intake. The SLA arm exhibited a positive change with regard to a significant decrease in fruit intake and a marginally significant increase in dietary fiber intake but a negative change with regard to a significant decrease in vegetable intake. Although there was a trend toward greater improvement in dietary behaviors in the I-ACE arm than the SLA arm at 12 months, none of the differences reached statistical significance, with the exception of a marginally significant lower intake of added sugar in the I-ACE arm.

As the I-ACE software enabled collecting dietary intake data at each counseling session, we were able to evaluate the within-person change in dietary behaviors across the study visits in the experimental arm (Table 3). Significant changes in the desired direction for all dietary variables occurred during the most intensive phase of the intervention (from baseline to 3 months), during which there were monthly counseling sessions. After that, there was a regression toward baseline intakes and only the changes in added sugar and dietary fiber from baseline remained significant at 12 months.

**Table 3:** Within-group differences in dietary behaviors from baseline to 2, 3, 6, and 12 months for 25 Arab patients with type 2 diabetes mellitus in the Interactive Lifestyle Assessment, Counseling, and Education pilot trial study arm.

Dietary variable	Difference between intake at baseline and at:^a^							
	2 months		3 months		6 months		12 months	
	Mean (SE)	*P* value	Mean (SE)	*P* value	Mean (SE)	*P* value	Mean (SE)	*P* value
Added sugar (% of total energy)	−1.8 (0.6)	.008	−1.9 (0.7)	.02	−0.9 (0.9)	.34	−2.6 (1.0)	.03
Dietary fiber (g/1000 kcal)	3.7 (0.6)	<.001	3.9 (0.7)	<.001	3.4 (0.8)	<.001	2.7 (0.9)	.003
Fruit (portions/day)	−0.7 (0.2)	.008	−0.7 (0.3)	.048	−0.5 (0.4)	.29	−0.4 (0.4)	.30
Vegetables (portions/day)	1.5 (0.2)	<.001	1.5 (0.3)	<.001	1.0 (0.4)	.02	0.1 (0.4)	.90
Whole grains (portions/day)	2.0 (0.4)	<.001	2.2 (0.5)	<.001	1.2 (0.6)	.09	−0.2 (0.6)	.75

^a^Multivariable linear mixed models for repeated measures controlling for sex. *P* value adjusted for multiple comparisons.

The increase in the percentage of participants in the I-ACE arm reporting any LPA (sex-adjusted odds ratio [OR] 2.80, 95% CI 0.67-11.58; *P*=.16) or the recommended LPA level (sex-adjusted OR 5.01, 95% CI 0.52-47.92; *P*=.16) was greater than that in the SLA arm (Multimedia Appendix 9). However, given the small sample size, the differences did not reach statistical significance.

For the repeated HbA_1c_ measurements, the model with natural splines with 2 knots (at 5 and 8 months) best fit the data. In addition, the final model included gender. The effect of the study arm was nonsignificant (*P*=.40); therefore, it was omitted from the model. The effect of time was significant (*P*<.001). According to the model, the mean HbA_1c_ values were expected to decrease from baseline levels by 11% after the 6-month follow-up (6 months/baseline HbA_1c_ ratio=0.89, 95% CI 0.88-0.91) and by 7% after the 12-month follow-up (12 months/baseline HbA_1c_ ratio=0.93, 95% CI 0.92-0.95). For example, the average HbA_1c_ level at baseline of 9.1% (95% CI 8.8-9.5) was expected to decline to 8.1% (95% CI 8.0%-8.2%) at the 6-month follow-up point and to 8.5% (95% CI 8.4%-8.6%) at the 12-month follow-up point.

**Figure 3:**
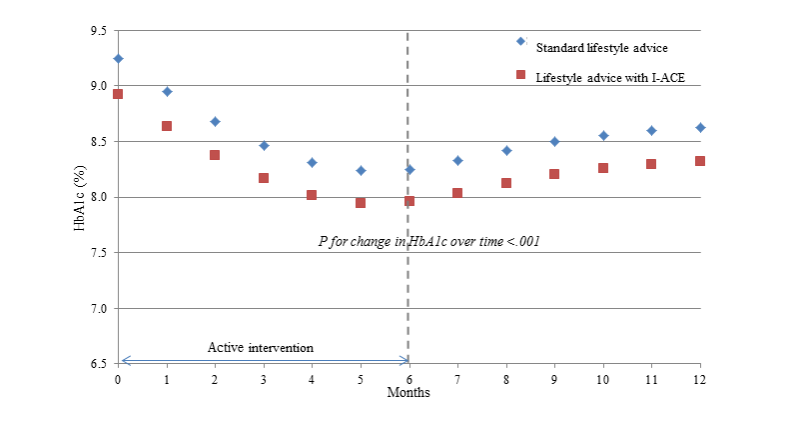
Expected HbA_1c_ values over time among 50 Arab patients with type 2 diabetes mellitus in the I-ACE pilot trial by study arm. Results from a linear mixed regression model for repeated measures, controlling for sex. HbA_1c_: hemoglobin A1c; I-ACE: Interactive lifestyle Assessment.

Figure 3 presents the predicted HbA_1c_ (in original units) for a typical subject from the SLA and I-ACE study arms. For the purpose of this figure, the study arm was not omitted from the model, although it was nonsignificant. HbA_1c_ levels decreased over time during the active intervention period (1-6 months) for both study arms, reaching a minimum value at approximately 6 months. During the postintervention follow-up period, the levels slightly increased but were still lower at 12 months than baseline levels. There was no significant change in WC over time and this did not differ by study arm in multivariable analysis. Crude results were similar for BMI, and owing to the nonnormal distribution of this variable and its close correlation with WC, multivariable analysis was not conducted for BMI.

**Figure 4:**
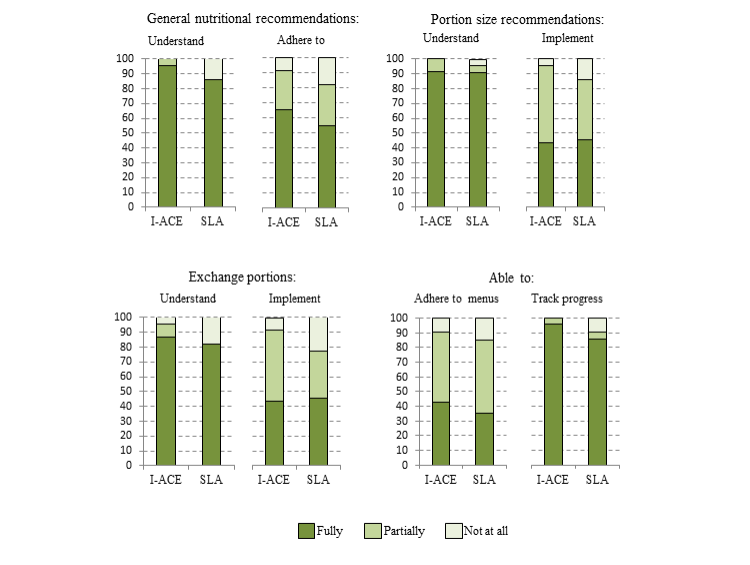
Participant responses to counseling utility questions regarding their ability to understand material and/or adhere to recommendations in the I-ACE pilot trial by study arm. Abbreviations: I-ACE Interactive lifestyle Assessment, Counseling, and Education; SLA Standard Lifestyle Advice.

Over 90% of the participants in both study arms expressed high satisfaction with the dietician and lifestyle counseling. Most participants indicated that they understood and could implement/utilize the information provided on general nutritional recommendations, portion sizes, exchanges portions, sample menus, and their progress over time; however, the proportion who indicated that they could only understand or implement these materials partially, or not at all, tended to be higher in the SLA than the I-ACE arm (Figure 4).

Most participants in the I-ACE arm thought that the I-ACE software was helpful to the dietary counseling (91%) and did not detract from their interaction with the dietician (96%). The study dietician also expressed overall satisfaction with the I-ACE software. She found the pictorial educational materials and the quantitative nutrient information, provided when individually tailoring sample menus, particularly helpful. She observed that younger participants who were technology oriented engaged more with the software in the counseling sessions than older participants. She noted areas that the software did not address (eg, emotional distress of coping with multiple comorbidities or other personal issues and other emotional support issues that may impact lifestyle behaviors and the readiness to make lifestyle changes).

### Harms

We have no harms or unintended consequences to report.

## Discussion

### Principal Findings

This pilot trial provided indications that the use of the culturally adapted I-ACE software for dietician-delivered lifestyle counseling can increase the pace of acquiring DM-related lifestyle knowledge and showed a trend toward improving lifestyle behaviors (diet and LPA). It was further associated with improved HbA_1c_ results, but not to a greater extent than the dietician-delivered SLA.

This study adds to the body of evidence supporting the efficacy of dietician-delivered dietary counseling for improving dietary knowledge and behaviors and health outcomes in patients with T2DM [3,36]. Consistent with our findings in this study, IT-assisted interventions among T2DM patients have resulted in more rapid knowledge acquisition than non-IT-assisted interventions, particularly when they utilize a combination of provider contact and technology [4,36].

This also holds for ethnic/racial minority groups, particularly when the interventions are culturally adapted/specifically tailored for the target groups [37,38]. In this trial, both groups received written materials in Arabic and dietary counseling from a culturally and linguistically congruent dietician. The digital platform used in the I-ACE arm enabled additional enhancement of cultural adaptation and individual tailoring. The study dietician had access to pictures and infographics reflecting ethnic dietary habits and commonly consumed foods to support the counseling process, both for educational purposes and for creating individually tailored sample menus.

In addition, I-ACE facilitated the use of a broad range of behavioral change modeling techniques to enable active interaction and immediate feedback. These included motivational interviewing, goal setting, goal modification, simulation of the effects of specific lifestyle changes, and tracking of progress. The Alive! IT-based lifestyle intervention, conducted in a workplace rather than specifically among T2DM patients, employed a similarly broad suite of behavioral change techniques (including the less common component of simulation) and showed significant lifestyle change compared with a wait-listed control group [39,40].

As an additional clinical counseling support feature, I-ACE makes it feasible to collect and update quantifiable data on changes in dietary behaviors at each counseling session, providing a new resource for tracking behavioral change over time. Furthermore, the documentation of the visit record and the dietary counseling process provides a data resource for quality control and other organizational purposes.

The I-ACE tool differs from existing lifestyle IT tools/applications, which typically rely upon multiple administrations of *daily trackers* to collect lifestyle data, resulting in a heavy user burden, user fatigue, and disuse [41]. In addition, these tools/apps are generally used independently by patients, and thus, quantified, actionable information about dietary self-management may not be readily accessible and is not a documented component of the clinical visit record [42-48]. Numerous studies have shown lifestyle-related IT tools to be more effective when used in clinical settings with health care provider contact than when used independently [4,36,49].

Few apps developed specifically for T2DM self-management include the assessment of dietary/lifestyle habits. Those that have included this domain either (1) did so in a superficial and nonquantified manner and did not improve dietary behaviors [44] or (2) required detailed recording of daily dietary intake, which resulted in a very high user burden [50] and was thus one of the least used features of mobile T2DM support apps [45]. Other apps/Web-based IT tools included unidirectional messages from the app to the patient or noninteractive Web-based information that did not support any individual tailoring of dietary/lifestyle plans [51,52].

Users’ satisfaction with I-ACE was high, suggesting it may enhance the dietary counseling process. Responses of the I-ACE arm participants to the utility questions also suggest that it may have potential for optimizing the patient’s understanding of and adherence to general nutritional recommendations, portion size and exchange portion recommendations, individually tailored menus, and the tracking of progress over time.

Reviews of the literature show that dietary counseling, particularly among patients with T2DM, improves the glycemic control to the same magnitude as expected from introducing a new drug [3,36-38,53]. In this pilot trial, we found that a short, dietician-delivered dietary counseling series (eg, 4 sessions over the course of 6 months) produced a decrease of 1% point from baseline HbA_1c_ at the end of the active intervention period and a 0.5% decrease at 12 months (6 months after the active intervention ended), in a sample starting with poor glycemic control.

At the same time, the limited number of dietary consultations in this pilot trial did not appear to be adequate to ensure fully sustained improvements in dietary behaviors and glycemic control. Systematic reviews of diabetes self-management education and nutritional interventions among adults with T2DM have shown a higher success rate in interventions with over 10 hours of contact with providers [4]. Interventions that provided dietary counseling encounters for more than 6 months reported that the improvement and continued reduction of HbA_1c_ was maintained for up to 2 years [3].

This pilot study has several strengths and limitations. The study adds to the very limited literature on culturally adapting and testing IT tools for low-SES ethnic minority populations and suggests that such tools can improve the efficiency of DM-related dietary education. The RCT design adds also to the limited body of RCTs evaluating the IT-supported lifestyle and educational interventions in low-SES minority communities, which typically have disproportionately high T2DM rates and few well-designed studies targeting the key self-management domain of nutrition among T2DM patients.

Although the study was adequately powered to test the primary outcome of DM-related dietary knowledge, it had limited power to test the secondary outcomes. Nevertheless, we observed trends toward greater improvement in lifestyle behaviors in the I-ACE arm than the SLA arm. This is particularly impressive as, from the outset, we aimed to recruit a challenging group of patients who were overweight or obese and had poor glycemic control, suggesting that despite having free access to health care services and subsidized medications, their engagement with and benefit from existing health services was suboptimal. Our experience with the intervention indicated that adequate resources and maximum institutional flexibility and accessibility are needed to insure successful implementation, particularly in vulnerable, low-SES populations. Nevertheless, we observed reasonably good adherence to the study intervention, with a suggested trend of improved engagement among men in the I-ACE arm so that it equaled that of women; whereas in the SLA arm, the men’s engagement was lower than that of women. Given the small sample in our pilot RCT, these suggested trends should be confirmed in a larger efficacy trial.

We used the same dieticians to deliver dietary counseling in both study arms to eliminate the possibility of differences between study arms because of different dieticians. The use of the I-ACE counseling approach may have affected the way the dietician delivered standard care in the comparison arm. In addition, the study was conducted in community clinics serving local neighborhoods comprised extended families. As the randomization was conducted on an individual basis, neighbors and extended family members who were assigned to different study arms may have had shared what they learned from the experimental intervention approach and materials outside of the clinic setting. Both these possible sources of contamination, however, would have led to an underestimation of the I-ACE experimental effect and thus do not detract from the significance of our findings. We recommend that a definitive RCT should consider a cluster randomized design, particularly if conducted in neighborhood clinics with very strong, extensive social networks.

Finally, the intervention in this pilot study was of limited duration and the findings suggest that a longer/more intensive intervention is important to assure sustainability.

### Generalizability

This study was conducted in a low-SES ethnic minority community at high risk of T2DM and its complications and showed potential for improving self-care, promoting healthy lifestyle behaviors, and improving surrogate health outcomes (HbA_1c_). The findings may be informative for planning definitive RCTs to evaluate IT-based clinical interventions in other similar populations.

The use of the I-ACE software took more time than is currently allocated in clinical care in Israel, particularly for the initial dietary counseling session (35-45 min), although the median time for follow-up visits (17 min) did not differ substantially from the 15-min slots allotted in routine follow-up care. This time differential, which is largely the result of the structured task flow, the additional tools at the dietician’s disposal, and the associated documentation, needs to be taken into account when considering application to clinical care. One possible accommodation would be to model routine clinical care differently. For example, the national health insurance basket of services in Israel includes 14 visits per year to a dietician for patients with DM, which, in practice, are rarely fully utilized. An alternative model worth testing would be the integration of I-ACE in a series of fewer but longer dietary counseling sessions, with a comparison of its effectiveness with the usual care with multiple short visits. In addition, further technological development, such as making the assessment and other features of the I-ACE software available for self-use by patients via mobile or Web apps, could reduce the time required for documentation in the face-to-face consultations with the dietician.

### Conclusions

This pilot trial supports the potential of the dietician-delivered, culturally adapted I-ACE dietary counseling intervention to increase the efficiency of DM-related lifestyle education and to improve lifestyle behaviors, compared with the usual dietary counseling in a minority population, although confirming that both types of counseling, coupled with active outreach, improved glycemic control. These findings provide support for conducting a large-scale trial to evaluate the effectiveness and applicability of the I-ACE software in routine clinical care among ethnically diverse populations.

## Appendix

Multimedia Appendix 1 CONSORT‐EHEALTH checklist (V 1.6.1).

Multimedia Appendix 2 Selected I-ACE screen shots.

Multimedia Appendix 3 Summary of the intervention delivery by study arm.

Multimedia Appendix 4 Examples of modification of diet knowledge questions.

Multimedia Appendix 5 DM-related lifestyle knowledge questionnaire. DM: diabetes mellitus.

Multimedia Appendix 6 Baseline DM diet-related knowledge scores among participants with and without data at 3, 6 and 12 months. DM: diabetes mellitus.

Multimedia Appendix 7 Number of counselling visits by study arm and qualitative information about intervention adherence.

Multimedia Appendix 8 Within-group and between-group differences in DM diet-related knowledge from baseline at 3, 6, and 12 months. DM: diabetes mellitus.

Multimedia Appendix 9 Difference in lifestyle behaviors at 12 months by study arm.

## Abbreviations

BMI: body mass index
DM: diabetes mellitus
FFQ: food frequency questionnaire
HbA_1c_: hemoglobin A1c
I-ACE: Interactive Lifestyle Assessment, Counseling, and Education
IQR: interquartile range
IT: information technology
LPA: leisure physical activity
OR: odds ratio
PA: physical activity
RCT: randomized controlled trial
SES: socioeconomic status
SKILLD: Spoken Knowledge in Low Literacy in Diabetes
SLA: standard lifestyle advice
T2DM: type 2 diabetes mellitus
WC: waist circumference
