# Supplementary material for: Development and Efficacy of an Electronic, Culturally Adapted Lifestyle Counseling Tool for Improving Diabetes-Related Dietary Knowledge: Randomized Controlled Trial Among Ethnic Minority Adults With Type 2 Diabetes Mellitus
Source: J Med Internet Res. 2019 Oct 16;21(10):e13674. doi: 10.2196/13674 (PMC6913526; doi:10.2196/13674)

Multimedia Appendix 2

Selected screen shots from the **I**nteractive lifestyle **A**ssessment, **C**ounseling and **E**ducation (**I-ACE**) software

- Food frequency questionnaire (FFQ) assessment screen
- Goal parameter screen
- Culturally-adapted educational infographics
- Simulation screen and personalized behavioral change take-home reports
- Follow-up visit assessment screen
- Reports tracking change over time

Screen shot of I-ACE Food Frequency Questionnaire* (FFQ)

1. Quantification of habitual dietary behaviors using an FFQ customized for Arabs with T2DM. Frequency of intake and number of portions are entered and average daily intake of energy and nutrients is immediately calculated and displayed.
2. Pop-up picture assists participants in reporting and shows a carbohydrate exchange portion for pita bread.

*English version; Arabic version used in counseling sessions

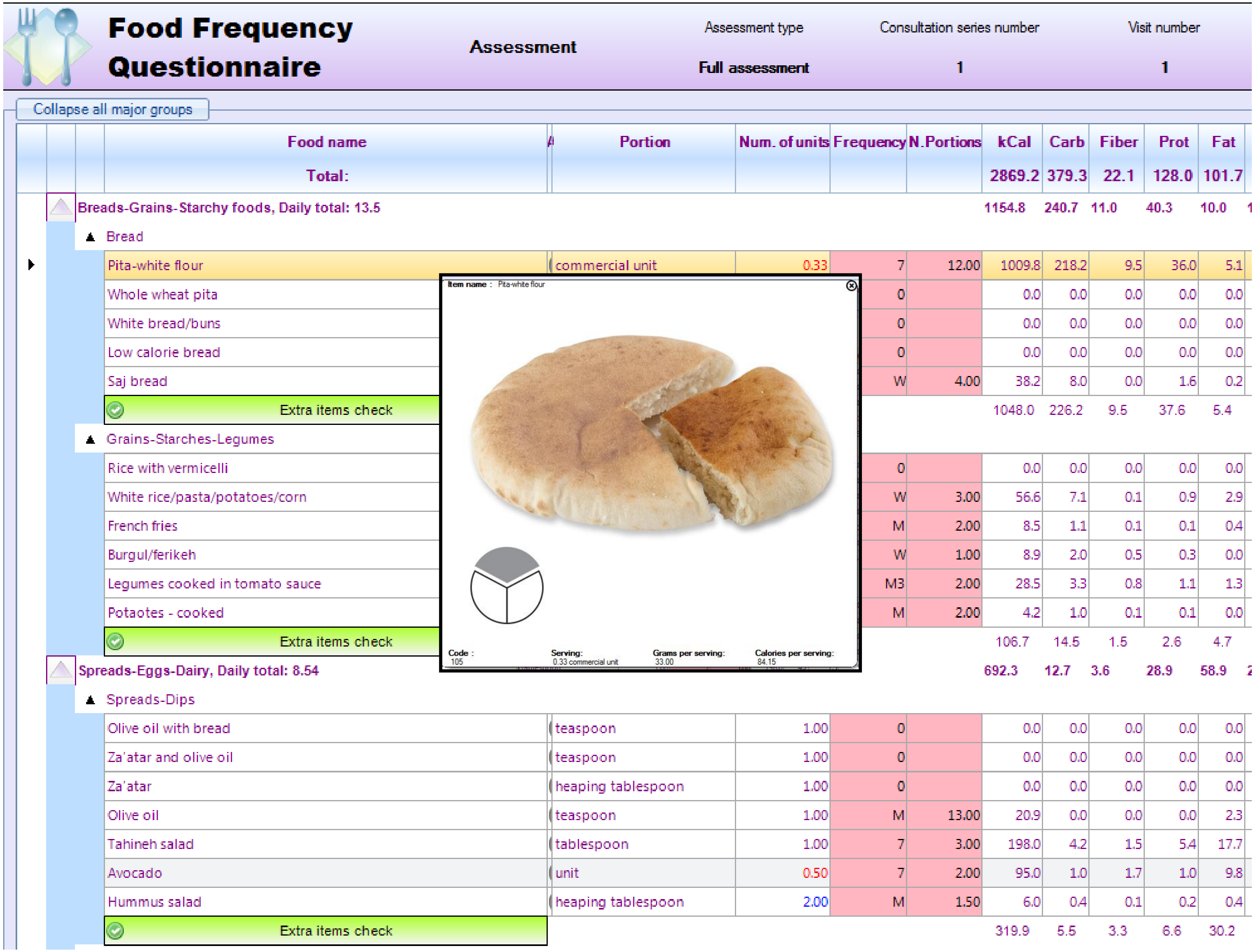

Food name	Portion	Num. of units	Frequency	N.Portions	kCal	Carb	Fiber	Prot	Fat
Total:					2869.2	379.3	22.1	128.0	101.7
Breads-Grains-Starchy foods, Daily total: 13.5					1154.8	240.7	11.0	40.3	10.0
Bread									
Pita-white flour	commercial unit	0.33	7	12.00	1009.8	218.2	9.5	36.0	5.1
Whole wheat pita	[illegible]	[illegible]	0		0.0	0.0	0.0	0.0	0.0
White bread/buns	[illegible]	[illegible]	0		0.0	0.0	0.0	0.0	0.0
Low calorie bread	[illegible]	[illegible]	0		0.0	0.0	0.0	0.0	0.0
Saj bread	[illegible]	[illegible]	W	4.00	38.2	8.0	0.0	1.6	0.2
Extra items check					1048.0	226.2	9.5	37.6	5.4
Grains-Starches-Legumes									
Rice with vermicelli	[illegible]	[illegible]	0		0.0	0.0	0.0	0.0	0.0
White rice/pasta/potatoes/corn	[illegible]	[illegible]	W	3.00	56.6	7.1	0.1	0.9	2.9
French fries	[illegible]	[illegible]	M	2.00	8.5	1.1	0.1	0.1	0.4
Burgul/ferikeh	[illegible]	[illegible]	W	1.00	8.9	2.0	0.5	0.3	0.0
Legumes cooked in tomato sauce	[illegible]	[illegible]	M3	2.00	28.5	3.3	0.8	1.1	1.3
Potaotes - cooked	[illegible]	[illegible]	M	2.00	4.2	1.0	0.1	0.1	0.0
Extra items check					106.7	14.5	1.5	2.6	4.7
Spreads-Eggs-Dairy, Daily total: 8.54					692.3	12.7	3.6	28.9	58.9
Spreads-Dips									
Olive oil with bread	teaspoon	1.00	0		0.0	0.0	0.0	0.0	0.0
Za'atar and olive oil	teaspoon	1.00	0		0.0	0.0	0.0	0.0	0.0
Za'atar	heaping tablespoon	1.00	0		0.0	0.0	0.0	0.0	0.0
Olive oil	teaspoon	1.00	M	13.00	20.9	0.0	0.0	0.0	2.3
Tahineh salad	tablespoon	1.00	7	3.00	198.0	4.2	1.5	5.4	17.7
Avocado	unit	0.50	7	2.00	95.0	1.0	1.7	1.0	9.8
Hummus salad	heaping tablespoon	2.00	M	1.50	6.0	0.4	0.1	0.2	0.4
Extra items check					319.9	5.5	3.3	6.6	30.2

Screen shot of selected goal parameters after lifestyle assessment*

1. Graphic comparison of reported leisure physical activity (PA) and selected nutrient/food group intakes, represented by 'current status' figure, to recommended levels/ranges (green zones)
2. Ideal goals/ranges are displayed, and personally-tailored interim goals may be set and modified.

*English version; Arabic version used in counseling sessions

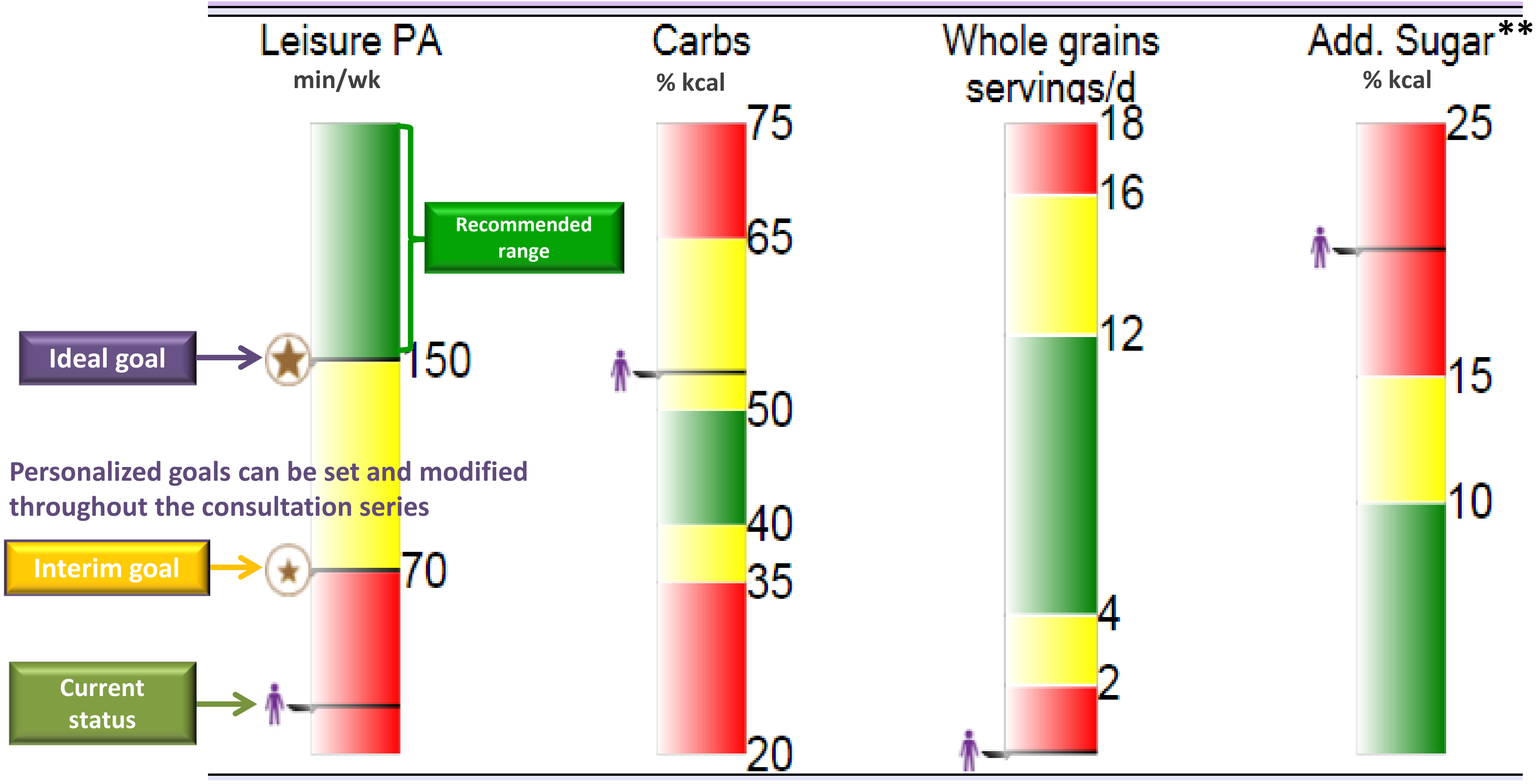

**Added sugar

Excerpts of culturally-adapted, Arabic-language infographics

Whole grains

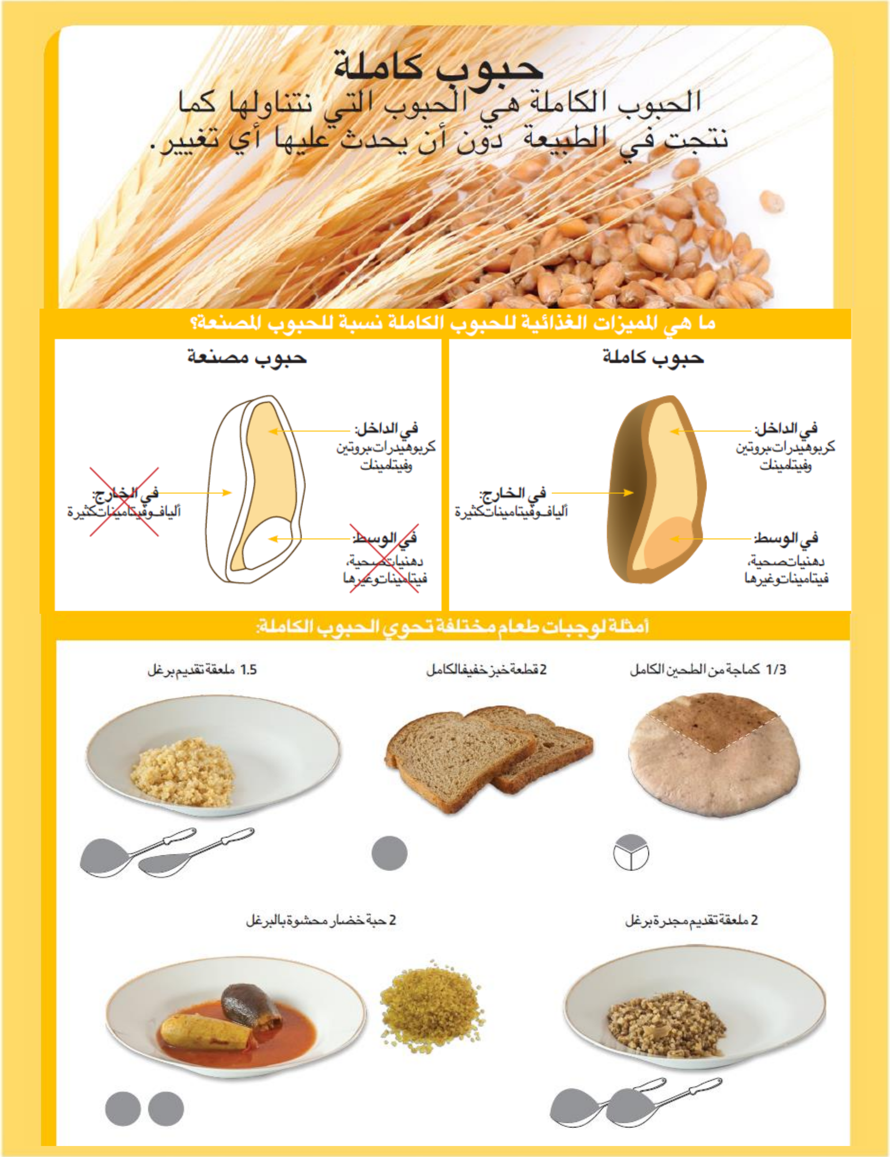

Physical activity

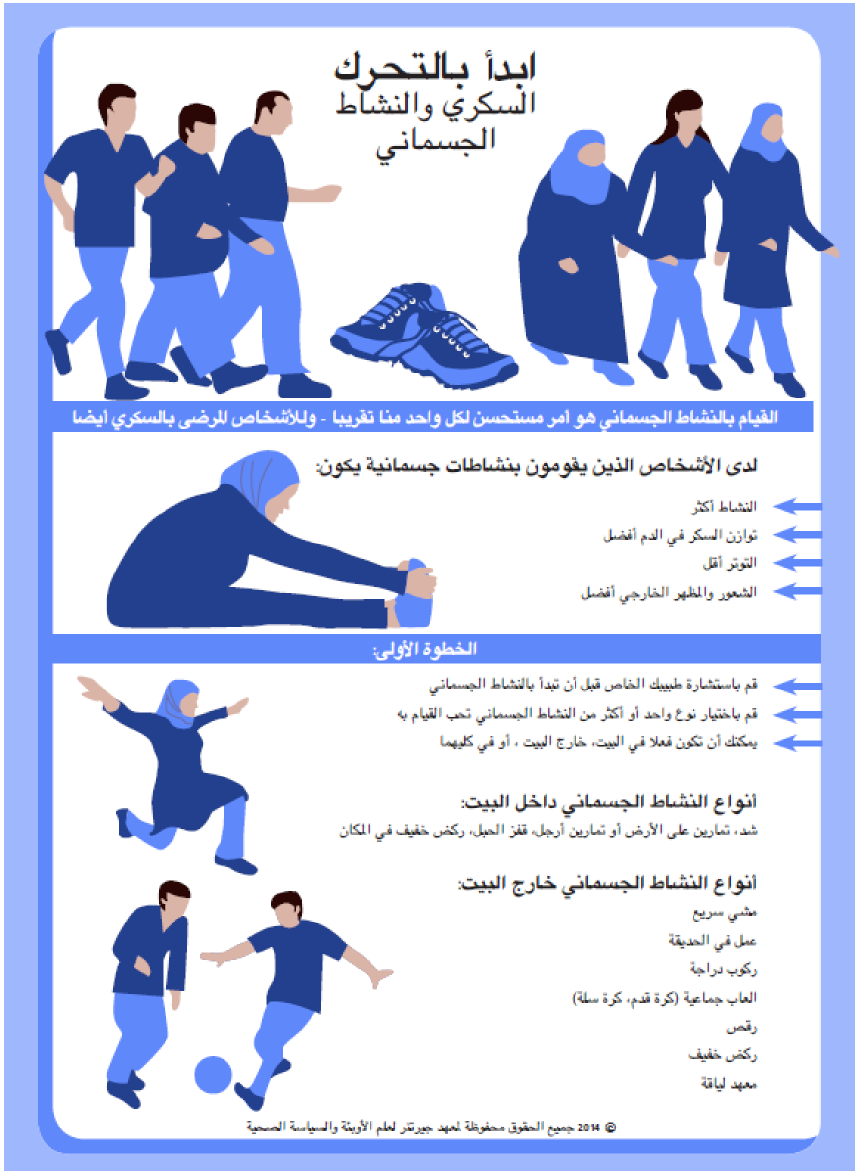

Excerpt of a culturally-adapted, Arabic-language carbohydrate exchange portion infographic showing the serving size of various types of breads that represents 1 carbohydrate exchange portion.

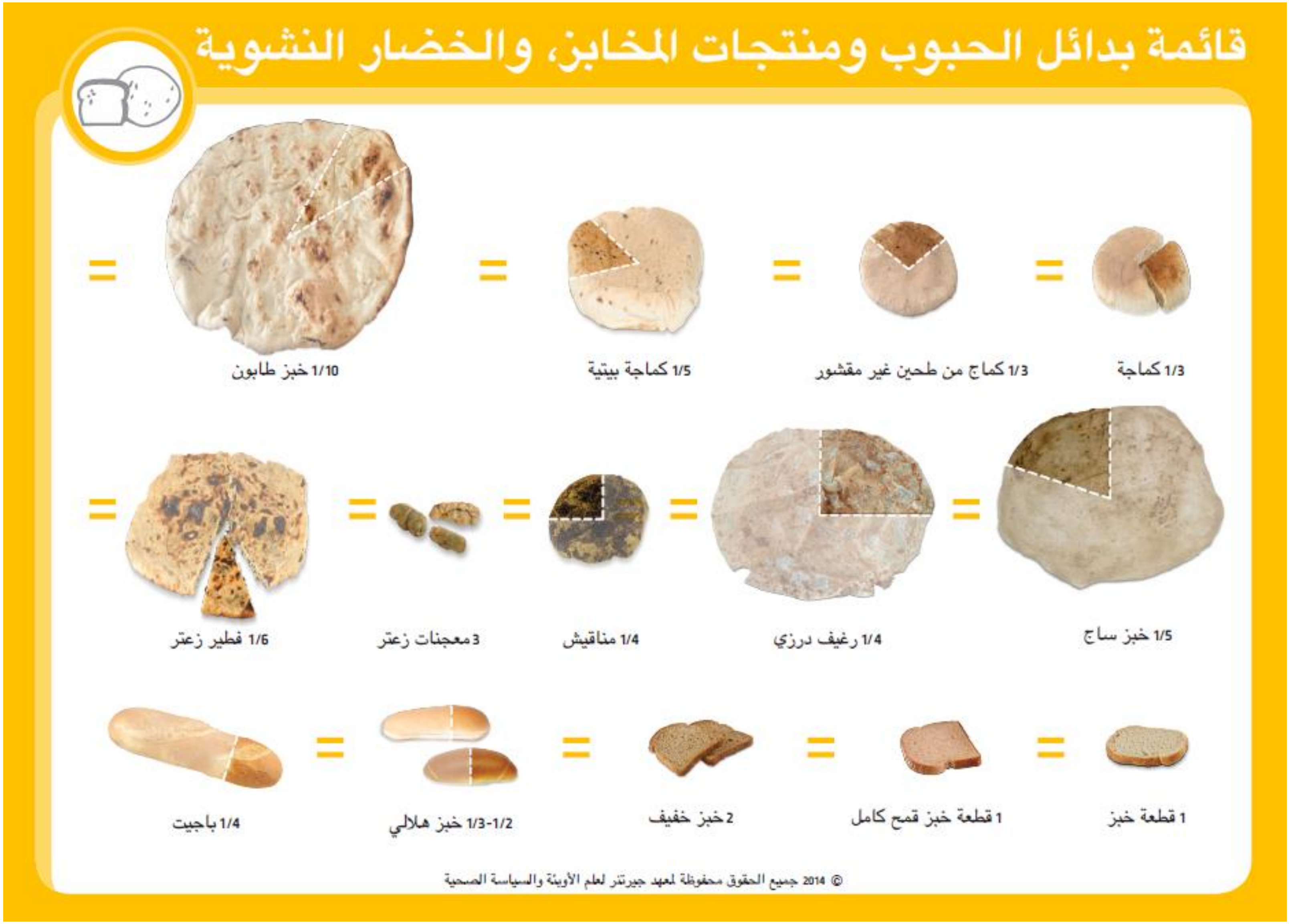

Screen shots from simulation and personalized behavioral change plan building

Excerpt of FFQ simulation screen and goal parameter for whole grains, showing patient status at assessment ( ), and after simulating changes to increase whole grain intake ( ).*

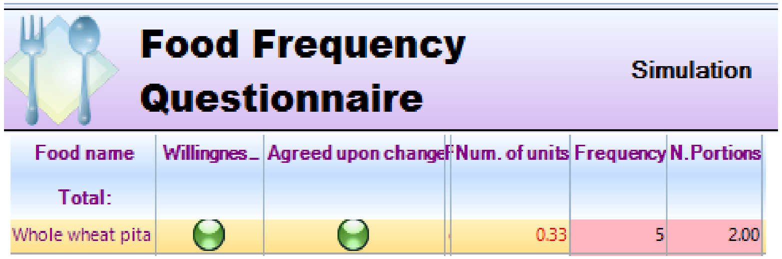

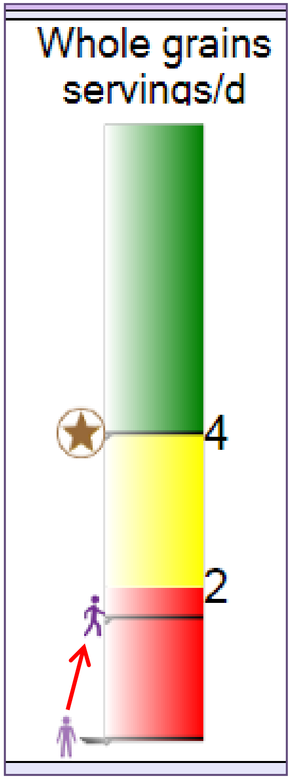

Excerpts of take-home reports* after completion of FFQ simulation of and agreement upon dietary changes

SUMMARY OF CHANGES

				CHANGE			
				FROM:		TO:	
				FFQ Assessment		FFQ Simulation	
Visit number/ date	Food item	Portion	# units in portion	Frequency	# portions	Frequency	# portions
1: 17/12/14	Pita bread, white flour	Store-bought Unit	0.33	7 d/wk	6	**0**	
	Pita bread, whole wheat	Store-bought Unit	0.33	0		**7 d/wk**	**6**
	Vegetable salad	Cup (200ml)	0.5	7 d/wk	2	**7 d/wk**	**5**
	Fresh fruit	Exchange portion	1	7 d/wk	4	**7 d/wk**	**2**
	Sweetend soft drinks	Cup (200ml)	0.5	7 d/wk	4	**1 d/wk**	**1**

*English version; Arabic version used in counseling sessions

Screen shot of I-ACE follow-up visit FFQ*

1. Follow-up visits build on the foundation of the assessments and simulations conducted in previous visits.
2. The follow-up assessment focuses on the patient's success with making previously agreed-upon changes (highlighted by a green 'agreed upon change' indicator), and the bulk of the visit focuses on further individually-tailored education and continued crafting of a personalized, stepwise, mutually agreed-upon plan for behavioral change.

*English version; Arabic version used in counseling sessions

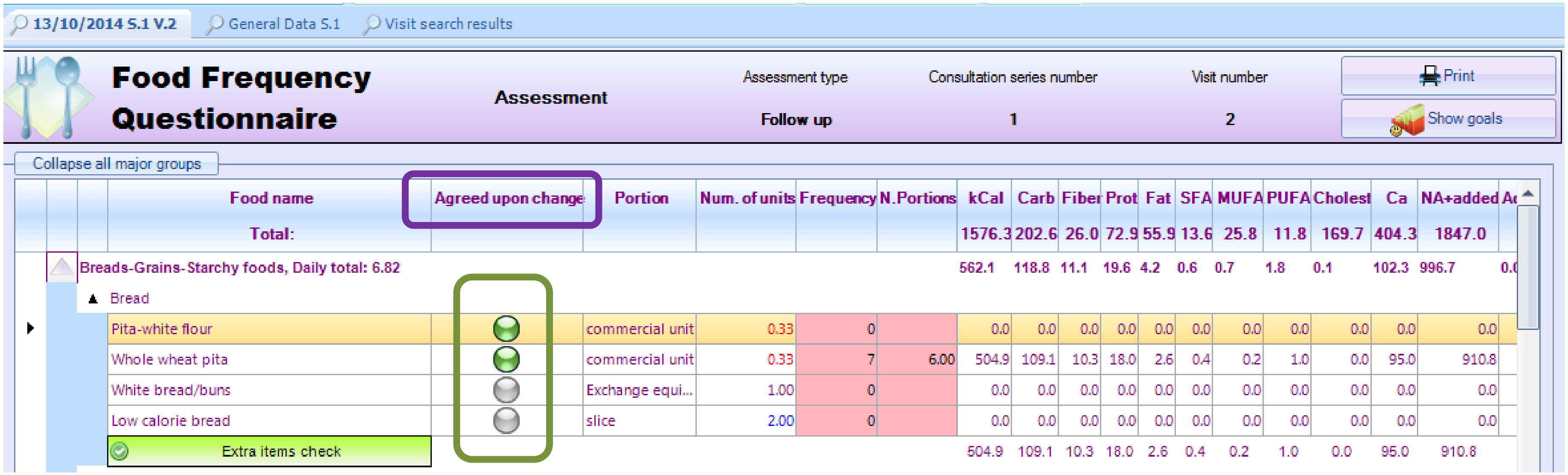

Food name	Agreed upon change	Portion	Num. of units	Frequency	N.Portions	kCal	Carb	Fiber	Prot	Fat	SFA	MUFA	PUFA	Cholest	Ca	NA+added	A[illegible]
Total:						1576.3	202.6	26.0	72.9	55.9	13.6	25.8	11.8	169.7	404.3	1847.0	
Breads-Grains-Starchy foods, Daily total: 6.82						562.1	118.8	11.1	19.6	4.2	0.6	0.7	1.8	0.1	102.3	996.7	0.[illegible]
Bread																	
Pita-white flour		commercial unit	0.33	0		0.0	0.0	0.0	0.0	0.0	0.0	0.0	0.0	0.0	0.0	0.0	
Whole wheat pita		commercial unit	0.33	7	6.00	504.9	109.1	10.3	18.0	2.6	0.4	0.2	1.0	0.0	95.0	910.8	
White bread/buns		Exchange equi...	1.00	0		0.0	0.0	0.0	0.0	0.0	0.0	0.0	0.0	0.0	0.0	0.0	
Low calorie bread		slice	2.00	0		0.0	0.0	0.0	0.0	0.0	0.0	0.0	0.0	0.0	0.0	0.0	
Extra items check						504.9	109.1	10.3	18.0	2.6	0.4	0.2	1.0	0.0	95.0	910.8	

I-ACE reports tracking change over time: Graphs of individual I-ACE arm participants' reported change across the 4-session active intervention and 12 mo. follow-up evaluations

Assessment value | **Simulation value** | **End of active intervention**

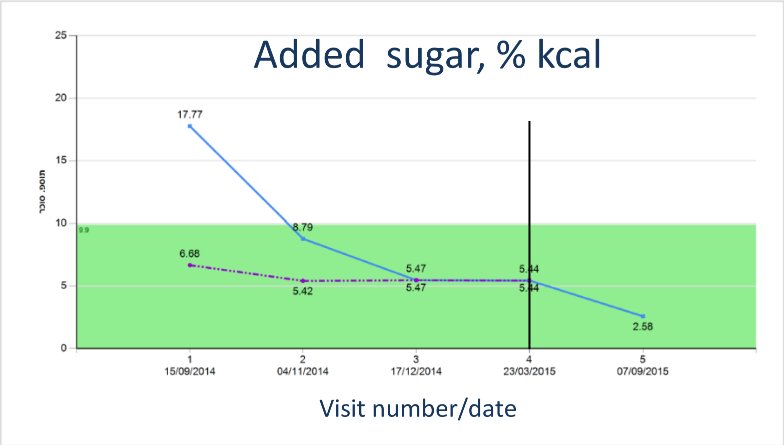

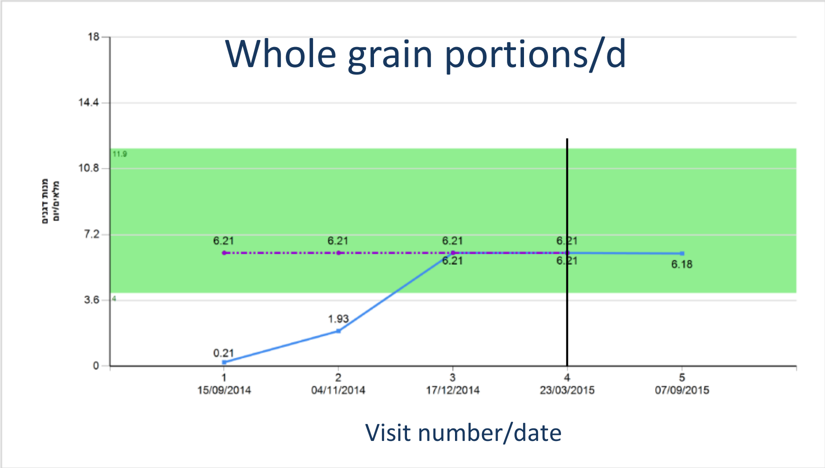

Fruit portions/d

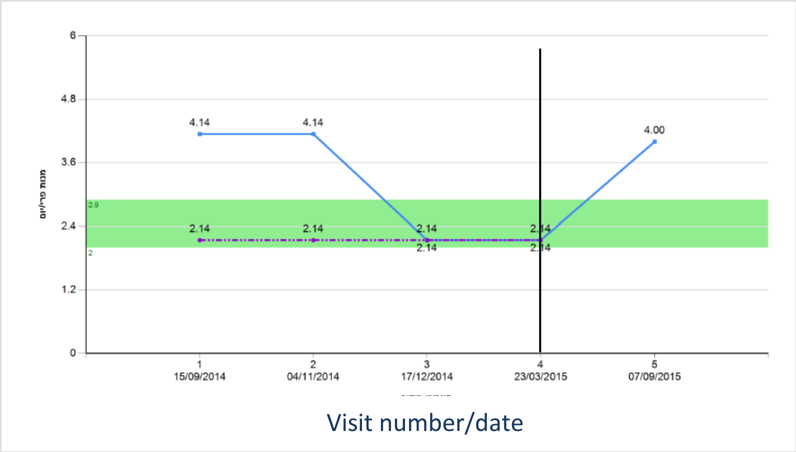

Vegetable portions/d

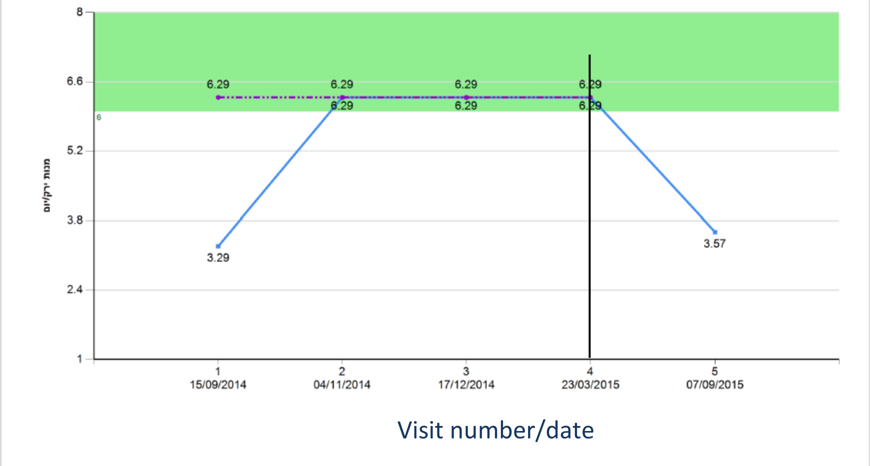

Supplement: Multimedia Appendix 2 [file jmir_v21i10e13674_app2.pdf]
