# Supplementary material for: Development and Efficacy of an Electronic, Culturally Adapted Lifestyle Counseling Tool for Improving Diabetes-Related Dietary Knowledge: Randomized Controlled Trial Among Ethnic Minority Adults With Type 2 Diabetes Mellitus
Source: J Med Internet Res. 2019 Oct 16;21(10):e13674. doi: 10.2196/13674 (PMC6913526; doi:10.2196/13674)
Supplement: Multimedia Appendix 3 [file jmir_v21i10e13674_app3.pdf]

Multimedia Appendix 1. Intervention components and activities by study arm for a pilot trial of a culturally-adapted lifestyle counseling IT[a] tool among 50 Arab participants with T2DM[b]

	**I-ACE[c] Arm**	**SLA[d] Arm**
Pre-randomization	Screening and baseline measurements	Screening and baseline measurements
Randomization		
Months 1-3	*Initial counselling session with I-ACE[c] tools*<br>2 follow-up counselling sessions with I-ACE tools<br>Diabetes education class with nurse educator	*Initial counselling session using SLA[d] methods*<br>2 follow-up counselling sessions with standard tools<br>Diabetes education class with nurse educator
Month 6	*Final counselling session with I-ACE[c] tools & data collection*	*Final counselling session with SLA[d] tools and data collection*
Month 12	*Final follow-up evaluation & data collection*	*Final follow-up evaluation & data collection*

[a]IT Information technology

[b]T2DM Type 2 diabetes mellitus

[c]I-ACE Interactive lifestyle Assessment, Counseling and Education

[d]SLA Standard Lifestyle Advice
