# Supplementary material for: Development and Efficacy of an Electronic, Culturally Adapted Lifestyle Counseling Tool for Improving Diabetes-Related Dietary Knowledge: Randomized Controlled Trial Among Ethnic Minority Adults With Type 2 Diabetes Mellitus
Source: J Med Internet Res. 2019 Oct 16;21(10):e13674. doi: 10.2196/13674 (PMC6913526; doi:10.2196/13674)
Supplement: Multimedia Appendix 4 [file jmir_v21i10e13674_app4.pdf]

Multimedia Appendix 4. Examples of modification of DM-related diet knowledge questions (* indicates correct answer) for a pilot trial of a culturally-adapted lifestyle counseling IT[a] tool among 50 Arab participants with T2DM[b]

Original question (source)	Modified question
Which of the following is highest in carbohydrate? (24)<br>1. Baked chicken<br>2. Swiss cheese<br>3. Baked potato*<br>4. Peanut butter	Which of these foods has the highest carbohydrate content?<br>1. Chicken<br>2. Cheese<br>3. Bread*<br>4. Tahini (sesame seed paste)<br>5. Don't know
Which of the following foods is highest in fat? (24)<br>1. Low-fat (2%) milk*<br>2. Orange juice<br>3. Corn<br>4. Honey	Which of these foods has the healthiest type of fat?<br>1. Local cheese<br>2. Tahini (sesame seed paste)*<br>3. Chips<br>4. Chicken wings<br>5. Don't know

[a]IT Information technology

[b]T2DM Type 2 diabetes mellitus
