# Supplementary material for: Development and Efficacy of an Electronic, Culturally Adapted Lifestyle Counseling Tool for Improving Diabetes-Related Dietary Knowledge: Randomized Controlled Trial Among Ethnic Minority Adults With Type 2 Diabetes Mellitus
Source: J Med Internet Res. 2019 Oct 16;21(10):e13674. doi: 10.2196/13674 (PMC6913526; doi:10.2196/13674)
Supplement: Multimedia Appendix 5 [file jmir_v21i10e13674_app5.pdf]

Multimedia Appendix 5: Diabetes-related lifestyle knowledge questionnaire for use among Arabs with type 2 diabetes mellitus in Israel (* indicates correct answer)

Are people with diabetes allowed to consume the following items? (check appropriate column)

Food/beverage	No, not at all	Only when blood sugar is too low	Yes, in limited amounts	Yes, without limits
1. Honey		*		
2. Dates			*	
3. Yogurt/buttermilk			*	
4. Regular cola (not diet)		*		
5. Vegetable salad				*
6. Rice			*	
7. Pita/bread			*	
8. Cookies			*	
9. Grapes			*	
10. Natural fruit juice		*		
11. Special foods for diabetics, without added sugar (e.g., cookies, chocolates)			*	

12. Which of these foods has the highest carbohydrate content?
    a. chicken
    b. cheese
    c. bread*
    d. sesame paste (tahini)
    e. don’t know

13. Which of these foods has the healthiest type of fat?
    a. locally-made cheese
    b. sesame paste (tahini)*
    c. deep-fried potatoes
    d. chicken wings
    e. don’t know

14. Which of the following foods/beverages causes blood sugar levels to rise the fastest?
    a. diet cola
    b. grapes*
    c. chicken
    d. pita bread
    e. don’t know

15. Which of the following foods/beverages causes the slowest rise in blood sugar levels?
    a. honey
    b. white bread
    c. bulgur*
    d. regular cola

What is the standard portion size for the following foods? *(*correct response)*

Food	Portion size	Don’t know
16. Pita bread	|__|__|.|__|__| units *(*0.25-0.33)*	|__|
17. Rice	|__|__|.|__|__| spoons *(*3)* /serving spoons *(*1)*	|__|
18. Apple	|__|__|.|__|__| units *(*1)*	|__|
19. Yogurt (natural)	|__|__|.|__|__| cups *(*1)*	|__|
20. Dried dates	|__|__|.|__|__| units *(*1)*	|__|

21. Which food/beverage can be used to raise blood sugar the most quickly during a hypoglycemic episode (when the blood sugar level is too low)?
    a. pita bread
    b. water
    c. cake/cookie
    d. regular cola/sugar-sweetened soft drink*

22. What is the effect of physical activity on blood sugar levels?
    a. lowers the blood sugar level*
    b. raises the blood sugar level
    c. has no effect

Are the following statements true or false?	True	False
23. The only way to achieve good blood sugar control is by eating primarily special foods for diabetics (e.g., foods without added sugar).	|__|	|*|
24. Reducing salt intake can help to reduce blood pressure.	|*|	|__|
25. Eating less animal fat (saturated fat) can help reduce the risk for heart disease.	|*|	|__|
