# Supplementary material for: Development and Efficacy of an Electronic, Culturally Adapted Lifestyle Counseling Tool for Improving Diabetes-Related Dietary Knowledge: Randomized Controlled Trial Among Ethnic Minority Adults With Type 2 Diabetes Mellitus
Source: J Med Internet Res. 2019 Oct 16;21(10):e13674. doi: 10.2196/13674 (PMC6913526; doi:10.2196/13674)
Supplement: Multimedia Appendix 6 [file jmir_v21i10e13674_app6.pdf]

Multimedia Appendix 6. Baseline DM[a] diet-related knowledge scores among participants with and without data at 3, 6 and 12 month follow-up assessments in a pilot trial of a culturally-adapted lifestyle counseling IT[b] tool among 50 Arab participants with T2DM[c]

Study assessment (months)	Baseline score for those without data		Baseline score for those with data		
	n	Mean±SD	n	Mean±SD	***P***
3	17	43.0±11.7	33	45.1±10. 6	.531
6	24	44.3±10.2	26	44.54±11.6	.949
12	5	38.8±8.1	45	45.0±11.0	.227

[a]DM diabetes mellitus

[b]IT Information technology

[c]T2DM Type 2 diabetes mellitus
