# Supplementary material for: Development and Efficacy of an Electronic, Culturally Adapted Lifestyle Counseling Tool for Improving Diabetes-Related Dietary Knowledge: Randomized Controlled Trial Among Ethnic Minority Adults With Type 2 Diabetes Mellitus
Source: J Med Internet Res. 2019 Oct 16;21(10):e13674. doi: 10.2196/13674 (PMC6913526; doi:10.2196/13674)
Supplement: Multimedia Appendix 7 [file jmir_v21i10e13674_app7.pdf]

Multimedia Appendix 7. Number of counseling visits by study arm in a pilot trial of a culturally-adapted lifestyle counseling IT[a] tool among 50 Arab participants with T2DM[b]

No. visits	Total n (%) Men		Women		*P*[c] .136	I-ACE[d] (n=25) Men		Women		*P*[e] .455	SLA[f] (n=25) Men		Women		*P*[g] .033	*P*[h] .450
1	5	(23.8)	2	(6.9)		0	(0.0)	2	(11.8)		5	(38.5)	0	(0.0)		
2	2	(9.5)	4	(13.8)		1	(12.5)	1	(5.9)		1	(7.7)	3	(25.0)		
3	6	(28.6)	4	(13.8)		3	(37.5)	3	(17.7)		3	(23.1)	1	(8.3)		
4	8	(38.1)	19	(65.5)		4	(50.0)	11	(64.7)		4	(30.8)	8	(66.7)		

[a]IT Information technology

[b]T2DM Type 2 diabetes mellitus

[c]*P* for Fischer's exact test for difference between men and women

[d]I-ACE Interactive lifestyle Assessment, Counseling and Education

[e]*P* for Fischer's exact test for difference between men and women in I-ACE arm

[f]SLA Standard Lifestyle Advice

[g]*P* for Fischer's exact test for difference between men and women in SLA arm

[h]*P* for Fischer's exact test for different between study arms

Qualitative information about adherence barriers

The study coordinator observed and reported that, regardless of study arm, people with T2DM in this low-SES minority community had to cope with many barriers and competing demands on their time. These included work and family obligations, lack of transportation, lack of availability during clinic hours, acute co-morbidities, etc. For some participants, these factors took priority over or hampered getting clinical support for their diabetes self-care, and participating in all intervention activities and evaluations.
