# Supplementary material for: Development and Efficacy of an Electronic, Culturally Adapted Lifestyle Counseling Tool for Improving Diabetes-Related Dietary Knowledge: Randomized Controlled Trial Among Ethnic Minority Adults With Type 2 Diabetes Mellitus
Source: J Med Internet Res. 2019 Oct 16;21(10):e13674. doi: 10.2196/13674 (PMC6913526; doi:10.2196/13674)
Supplement: Multimedia Appendix 8 [file jmir_v21i10e13674_app8.pdf]

Multimedia Appendix 8. Within-group and between-group differences[a] in DM[b] diet-related knowledge from baseline at 3, 6 and 12 months by study arm in a pilot trial of a culturally-adapted lifestyle counseling IT[c] tool among 50 Arab participants with T2DM[d]

Time (mos)	Difference in knowledge score from baseline within SLA[e] arm			Difference in knowledge score from baseline within I-ACE[f] arm			Difference in knowledge score between groups (slope)		
	**n**	**Mean±SE**	***P***	**n**	**Mean±SE**	***P***	**n**	**Mean±SE**	***P***
3	15	3.11±2.89	.288	18	11.92±2.76	<.001	33	8.80±3.99	.033
6	14	9.10±2.90	.003	12	8.74±2.92	.005	26	-0.35±4.11	.932
12	21	8.04±2.93	.009	24	8.29±2.81	.005	45	0.24±4.06	.954

[a]From a multivariable linear mixed model for repeated measures controlling for sex, educational level, baseline general DM knowledge (SKILLD)[30], and baseline HbA1c

[b]DM Diabetes mellitus

[c]IT Information technology

[d]T2DM Type 2 diabetes mellitus

[e]SLA Standard Lifestyle Advice

[f]I-ACE Interactive lifestyle Assessment, Counseling and Education
