# Supplementary material for: Development and Efficacy of an Electronic, Culturally Adapted Lifestyle Counseling Tool for Improving Diabetes-Related Dietary Knowledge: Randomized Controlled Trial Among Ethnic Minority Adults With Type 2 Diabetes Mellitus
Source: J Med Internet Res. 2019 Oct 16;21(10):e13674. doi: 10.2196/13674 (PMC6913526; doi:10.2196/13674)
Supplement: Multimedia Appendix 9 [file jmir_v21i10e13674_app9.pdf]

Multimedia Appendix 9. Difference in lifestyle behaviors at 12 months by study arm in a pilot trial of a culturally-adapted lifestyle counseling IT[a] tool among 50 Arab participants with T2DM[b]

A. **Dietary behaviors**

Dietary variable	I-ACE[c] arm Mean±SD			SLA[d] arm Mean±SD			P^g
	**Baseline (n=25)**	**12 months (n=24)**	P^e	**Baseline (n=25)**	**12 months (n=21)**	P^f	
Added sugar (% of total energy)	5.5±5.2	2.9±2.2	.036	5.4±5.8	4.9±4.5	.537	.050
Dietary fiber (g/1000 kcal)	9.7±2.6	12.4±3.4	.006	9.5±2.4	11.8±4.3	.055	.578
Fruit (portions/d)	3.0±1.7	2.6±1.5	.443	2.9±1.7	2.3±1.0	.003	.203
Vegetables (portions/d)	3.4±1.9	3.5±1.5	.902	4.2±2.4	3.1±1.2	.014	.172
Whole grains (portions/d)	2.2±2.7	2.0±2.0	.805	2.2±2.5	3.0±3.4	.579	.325

[a]IT Information technology

[b]T2DM Type 2 diabetes mellitus

[c]I-ACE Interactive lifestyle Assessment, Counseling and Education

[d]SLA Standard Lifestyle Advice

[e]*P* for univariable paired t-test for difference between baseline and 12 months in I-ACE arm

[f]*P* for univariable paired t-test for difference between baseline and 12 months in SLA arm

[g]*P* for multivariable linear regression models controlling for baseline intake and sex in all models for the 41 participants with dietary data at 12 mos. Other key demographic variables with $P<.10$ were controlled for in relevant models: the added sugar model also controlled for study city; and the vegetable and whole grain models also controlled for age.

B. Physical activity behaviors

Leisure physical activity	I-ACE[a]				SLA[b]				P[c]
	Baseline (n=25)		12 months (n=24)		Baseline (n=25)		12 months (n=21)		
	n	%	n	%	n	%	n	%	
Any	5	20.0	9	37.5	4	16.00	4	19.1	.205
Meeting recommended level (2.5+ h/wk)	2	8.0	5	20.8	2	8.0	1	4.8	.193

[a]I-ACE Interactive lifestyle Assessment, Counseling and Education

[b]SLA Standard Lifestyle Advice

[c]*P* for Fisher's Exact Test for the difference between study arms at 12 months
